# Reservoir Computing Properties of Neural Dynamics in Prefrontal Cortex

**DOI:** 10.1371/journal.pcbi.1004967

**Published:** 2016-06-10

**Authors:** Pierre Enel, Emmanuel Procyk, René Quilodran, Peter Ford Dominey

**Affiliations:** 1 Univ Lyon, Université Lyon 1, Inserm, Stem Cell and Brain Research Institute U1208, Bron, France; 2 Icahn School of Medicine at Mount Sinai, New York, New York, United States of America; 3 Escuela de Medicina, Departamento de Pre-clínicas, Universidad de Valparaíso, Hontaneda, Valparaíso, Chile; Oxford University, UNITED KINGDOM

## Abstract

Primates display a remarkable ability to adapt to novel situations. Determining what is most pertinent in these situations is not always possible based only on the current sensory inputs, and often also depends on recent inputs and behavioral outputs that contribute to internal states. Thus, one can ask how cortical dynamics generate representations of these complex situations. It has been observed that mixed selectivity in cortical neurons contributes to represent diverse situations defined by a combination of the current stimuli, and that mixed selectivity is readily obtained in randomly connected recurrent networks. In this context, these reservoir networks reproduce the highly recurrent nature of local cortical connectivity. Recombining present and past inputs, random recurrent networks from the reservoir computing framework generate mixed selectivity which provides pre-coded representations of an essentially universal set of contexts. These representations can then be selectively amplified through learning to solve the task at hand. We thus explored their representational power and dynamical properties after training a reservoir to perform a complex cognitive task initially developed for monkeys. The reservoir model inherently displayed a dynamic form of mixed selectivity, key to the representation of the behavioral context over time. The pre-coded representation of context was amplified by training a feedback neuron to explicitly represent this context, thereby reproducing the effect of learning and allowing the model to perform more robustly. This second version of the model demonstrates how a hybrid dynamical regime combining spatio-temporal processing of reservoirs, and input driven attracting dynamics generated by the feedback neuron, can be used to solve a complex cognitive task. We compared reservoir activity to neural activity of dorsal anterior cingulate cortex of monkeys which revealed similar network dynamics. We argue that reservoir computing is a pertinent framework to model local cortical dynamics and their contribution to higher cognitive function.

## Author Summary

One of the most noteworthy properties of primate behavior is its diversity and adaptability. Human and non-human primates can learn an astonishing variety of novel behaviors that could not have been directly anticipated by evolution. How then can the nervous system be prewired to anticipate the ability to represent such an open class of behaviors? Recent developments in a branch of recurrent neural networks, referred to as reservoir computing, begins to shed light on this question. The novelty of reservoir computing is that the recurrent connections in the network are fixed, and only the connections from these neurons to the output neurons change with learning. The fixed recurrent connections provide the network with an inherent high dimensional dynamics that creates essentially all possible spatial and temporal combinations of the inputs which can then be selected, by learning, to perform the desired task. This high dimensional mixture of activity inherent to reservoirs has begun to be found in the primate cortex. Here we make direct comparisons between dynamic coding in the cortex and in reservoirs performing the same task, and contribute to the emerging evidence that cortex has significant reservoir properties.

## Introduction

One of the properties that sets primates apart in the animal kingdom is their extraordinary adaptation skills which are supported by efficient context-dependent learning mechanisms. The ability to reliably encode unanticipated behavioral contexts appears to be crucial to such adaptive capabilities. Indeed, one of the most influential theories of prefrontal cortex (PFC) function states that in order to link sensory information to appropriate actions, the PFC must develop the relevant contextual representations, with a high capacity for multimodality and integration [1]. Although the remarkable representation capabilities in the activity of PFC areas have been explored in a wide variety of tasks, their origin is still unknown, as is the formidable capacity of the PFC to represent such diverse relevant situations.

Recently, Rigotti et al. [2] proposed that, rather than prewiring a network for the relevant representations needed for a task, activity in a randomly connected network could represent essentially all possible combinations of the task stimuli. The corresponding recombination of inputs observed in the activity of single units has been termed mixed selectivity, and its non-linear components are believed to support the representation of the conjunction of several stimuli. Although observed in early PFC studies while animals performed tasks involving multiple variables [3–6], mixed selectivity in the PFC has only recently become a specific research focus [7]. In the latter study, the authors demonstrated that these non-linear combinations of task variables were absent in PFC activity when monkeys made errors, emphasizing the importance of mixed selectivity in encoding behavioral context. This context can be defined not only with the current set of stimuli directly available from the environment, but also with previous stimuli and actions that define the internal state of the agent.

Interestingly, such network representations of arbitrary combinations of current and past inputs have been the focus of several research groups studying the recently baptized reservoir computing framework. Reservoirs are recurrent networks with fixed connections that are randomly generated according to certain parameters to obtain rich spatial (i.e. nature of inputs) and temporal (i.e. previous inputs, and their order and timing) representations composed of combinations of inputs. A simple linear output reads the activity of the recurrent network to extract meaningful representations. The first instantiation of reservoir computing was Dominey et al.'s temporal recurrent network model [8] of cortico-striatal function in sequence processing and production. With the PFC as the reservoir and the striatum as readout, the model provided an explanation of one of the first neurophysiological studies of mixed selectivity in PFC [4]. Barone and Joseph [4] identified neurons in the peri-arcuate oculomotor area whose responses encoded a mixture of spatial location and sequence rank, in an oculomotor sequencing task. In Dominey et al. [8], the recurrent PFC reservoir modeled a prevalent feature of cortical connectivity which is a strong local recurrence, and generated a mixture of spatial and sequential rank selectivity in the reservoir neurons, as observed in primate prefrontal cortex single units [4]. This reservoir computing paradigm was further developed independently by two teams in computational neuroscience [9] and machine learning [10, 11]. Maass et al. [9] developed a spiking neuron reservoir called the “liquid state machine” and demonstrated the universal computing power of this type of network, while Jaeger investigated the signal processing capacities of an analog reservoir called the “echo state network”. Remarkably, these spatio-temporal reservoir properties have also been found in primary cortical areas of monkeys and cats [12–15], as stimuli presented in the past influence the representation of subsequent stimuli. Furthermore, and importantly, *in vitro* randomly connected recurrent networks of cortical rat neurons display spatio-temporal processing similar to a reservoir [16].

The simplest architecture of the reservoir networks does not include feedback from output neurons to the recurrent network. Hence, the memory of previous inputs is only supported by recurrent connections that create loops in the connectivity of the network, yielding a dynamic system that allows past inputs to reverberate and influence the processing of current inputs. As a consequence, this classic architecture supports a fading memory of inputs. To circumvent this temporal limitation, Pascanu and Jaeger [17] demonstrated that output units, which feed back into the recurrent network and explicitly represent relevant information act as a working memory through an input driven attractor which can indefinitely hold this information in memory. Similarly, Maass and colleagues demonstrated the simultaneity of attracting dynamics and real-time computing in reservoirs with feedback units, thereby expanding the computational power of reservoirs [18, 19]. This feedback mechanism would allow the representation of task related contexts that are defined by current/recent inputs and contextual information that span longer time periods than the limited fading memory of a classic reservoir.

Pascanu and Jaeger [17] thus note the crucial distinction between attractors in autonomous systems, vs. input-driven systems. They introduce a mechanism whereby memory states intuitively correspond to attractors in an input driven system. Memory is implemented by neurons that are trained to lock into an on state when the remembered item appears. This on state is reinjected into the reservoir thus creating modified attractor state. They demonstrate this in scenarios where the memory task is to keep track of bracket nesting in a sequence of characters, and the ongoing task is to predict the next character, which varies depending on the bracketing level. Six WM units allow up to six levels of bracketing to be represented. The activity of these WM units feeds back into the reservoir thus creating different attractors, such that the reservoir behaves differently in the prediction task depending on which attractor it is in. Pascanu and Jaeger [17] note that a similar switchable system was demonstrated by Sussillo and Abbott [20] via learning that operated simultaneously on reservoir and readout to yield robust submodes of reservoir dynamics.

In the present study, we present a proof of concept of the reservoir computing framework to model information representation schemes and neural dynamics properties of the PFC. With a reservoir, we modeled a complex cognitive task initially developed for monkeys and explored neural activity representations at both the single unit and population levels. Non-linear mixed selectivity was inherently present in the network as was a dynamic mixed-selectivity that related to temporal information. In the memory version of the model, a feedback neuron explicitly representing behavioral context created a hybrid dynamical regime with two input driven attractor states that were induced by the behavioral context in memory allowing the system to process stimuli and perform the task in a context dependent manner. These experiments demonstrate the spatio-temporal processing capacities of reservoir networks in both situations—with and without explicit context feedback—in the context of a cognitive task originally developed for monkeys. In order to compare reservoir and cortical activity and dynamics, we used similar analyses on dorsal anterior cingulate cortex (dACC) activity from monkeys that performed the same cognitive task. We previously demonstrated that the dACC [21] and dorsolateral prefrontal cortex [22] play complimentary roles in this task [23]. dACC plays a greater role than DLPFC in integration of positive and negative feedback and tracking exploration vs. exploitation phases of the task. DLPFC activity was more tightly related to monkeys’ behavior than dACC activity, displaying higher mutual information with animals’ choices than dACC. dACC thus displays rich activity related to behavioral feedback, exploration vs. exploitation and behavior selection. We show that both representational and dynamical features present in the reservoir are observed in this prefrontal area, further validating the reservoir computing framework as a relevant approach to understand information processing and representation in the PFC.

## Materials and Methods

### Ethics statement

All procedures were carried out according to the 1986 European Community Council Directives (86/609/EEC), the French Ministère de l’Agriculture et de la Forêt, French Commission of animal experimentation, the Department of Veterinary Services (DDSV Lyon, France). At the time of the experiments authorization was granted under regional rules to the laboratory for a range of experiments, rather than for specific studies. Specific authorization covering this study was delivered by the ‘‘Préfet de la Région Rhône Alpes” and the ‘‘Directeur départemental de la protection des populations” under Permit Number: #A690290402, including approved protocols in NHPs (#047, #048, #0198, #0199, #0200). All procedures complied with guidelines for animal welfare in accordance with the recommendations of the Weatherall report, ‘‘The use of non-human primates in research”.

### Problem solving task

**Fig 1:**
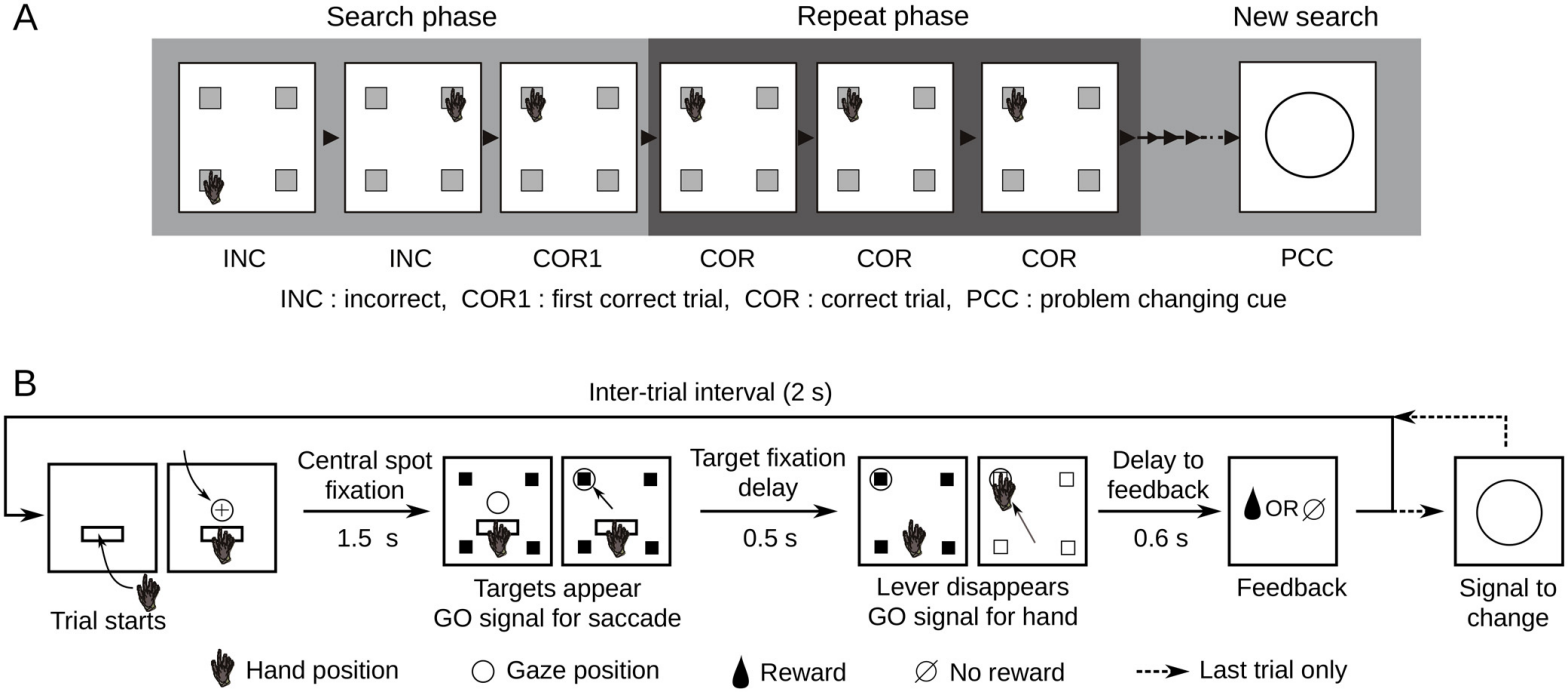
Problem solving task. **A**. An example problem. Monkeys searched through the targets by trial and error to find which target was rewarded. Incorrect trials (INC) and first rewarded trial (COR1) constitute the search phase. In this example, the upper left target is rewarded. The set of subsequent rewarded trials is defined as the repeat phase, made up of correct (COR) trials. At the end of the repeat phase a problem changing cue indicated to the monkey that a new problem was starting, thus a new target was rewarded. **B**. Single trial events. Trials started with hand on lever and fixation of a central point. A 1.5s delay ensued before targets appeared and fixation point disappeared, providing the GO signal. The monkey made a saccade to the chosen target and fixated it for 0.5s. The GO signal for hand movement was given by lever removal and the monkey touched the fixated target. All targets go blank at the time of touch and disappeared at feedback. Feedback was preceded by a 0.6s delay and followed by a 2s inter-trial interval.

In order to compare the neural activity in the recurrent network model with that of the behaving primate cortex, we tested both systems using a problem solving task that was originally developed by Procyk and Goldman-Rakic [24] to investigate shifts between exploration and exploitation behavior (see Quilodran et al., [21] for detailed description). Two rhesus monkeys had to find by trial and error which among four targets presented on a touch screen was rewarded by fruit juice (Fig 1A). At the onset of a trial, monkeys fixated a central fixation point and held their hand on a lever displayed on the screen below the fixation point (Fig 1B). After a delay period of 1.5 seconds, 4 targets appeared on the screen. The animals made a saccade to one target and fixated it for 0.5 seconds until the lever disappeared, giving the GO signal to touch the chosen (fixated) target. Feedback was preceded by a 0.6-second delay, and followed by a 2-second delay ending at the beginning of next trial. The search phase included the incorrect trials (INC) and the first rewarded trial (COR1) during which animals explored the targets. The following 3 correct trials (COR) allowed the monkeys to repeat the rewarded choice and constituted the repetition phase. Occasionally (10% of cases) repetition lasted for 7 or 11 trials to prevent the animals from anticipating the end of the repetition phase. A signal to change appeared at the end of the last repetition trial to indicate to the animal that a new target was going to be rewarded.

A search phase and its following repeat phase are referred to as a problem. In only 10% of cases the same target was rewarded in two consecutive problems. After training, monkeys performed the task in a nearly optimal fashion. In each search, they avoided previously explored targets that were not rewarded, and correctly repeated the rewarded choice. Likewise, they generally avoided repeating the previously rewarded target in the subsequent problem. The average number of trials in search was 2.4 ± 0.15 for first monkey and 2.65 ± 0.23 for second monkey (knowing that the same target is not rewarded two problems in a row, the expected number of trials of an optimal search is ~2.2) and in repetition 3.14 ± 0.7 and 3.4 ± 0.55 for first and second monkey respectively (the optimal-repetition trial number is above 3, as some problems had more than 3 rewarded repetition trials).

### Recurrent neural network model

We developed a recurrent neural network model using reservoir computing (RC) to perform the cognitive task in order to generate predictions that could then be tested with dACC monkey data. According to the RC principle, a fixed, large, random reservoir (recurrent neural network RNN) is excited by input signals, and the desired output is combined from the excited reservoir signals by a trainable readout mechanism (a simple linear regression in the most simple versions). As mentioned, the RC principle has been independently discovered in cognitive neuroscience (temporal recurrent networks, [8, 25]), in computational neuroscience (liquid state machine, [9]), and in machine learning (echo state networks, [10]). Models have been recently developed along the RC principle to reproduce cognitive functions like working memory [17] and language comprehension and production [26–28]. Two versions of the model were used in order to obtain the results of this paper: the original version and a second version implementing a simple contextual memory. The initial version was used in single neuron analyses in the first part of the results while the contextual memory version was introduced later to show the benefits of context encoding [29].

**Fig 2:**
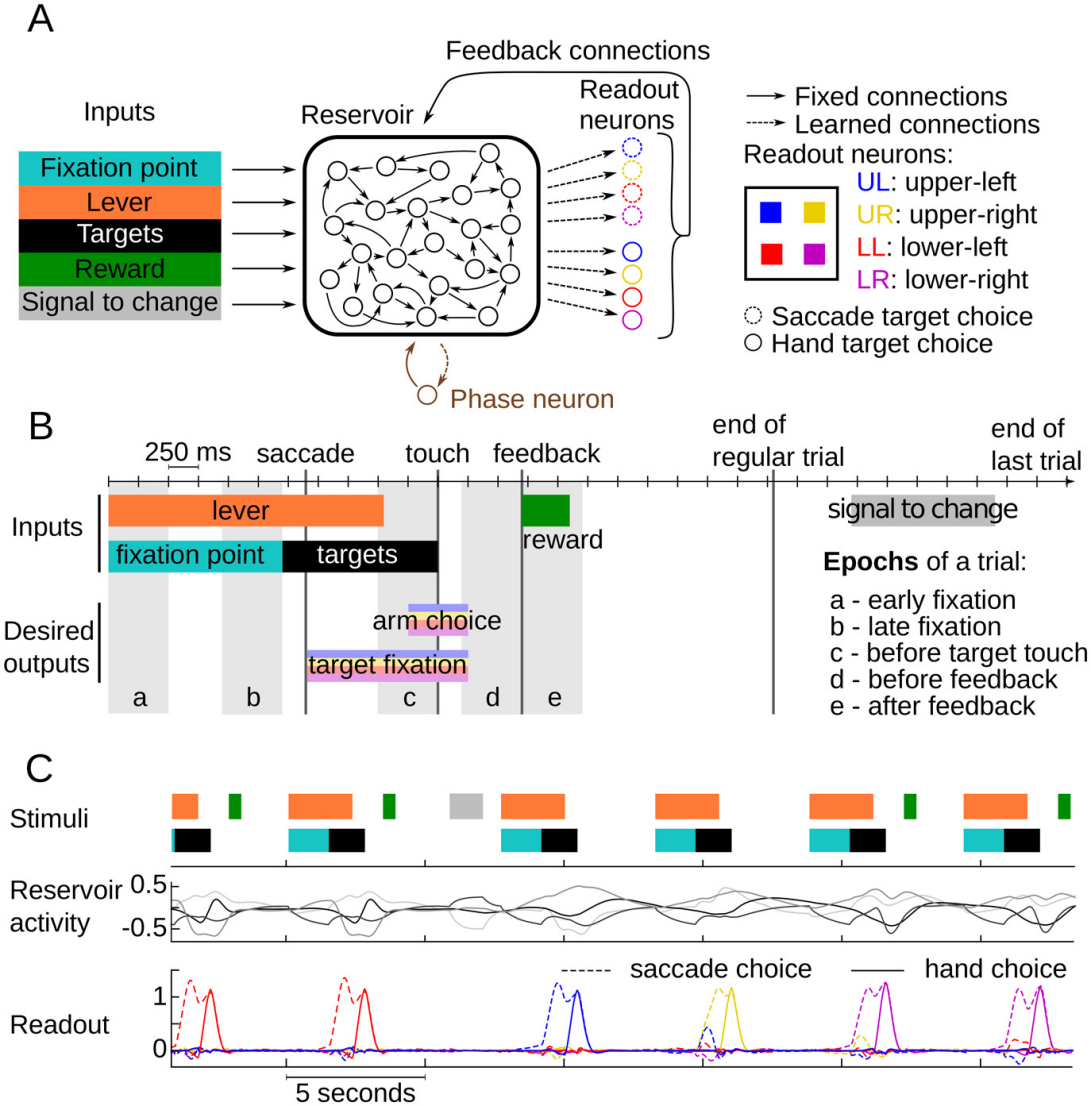
Model architecture, modeled task and test-activity example. **A**. Model architecture. A recurrent network of 1000 randomly connected neurons (the reservoir) received input from 5 units representing the presence of the fixation point, the lever, the targets, the reward and the signal to change. Output choice of the network was represented in two sets of 4 readout neurons corresponding to target fixation and arm touch respectively. Connections between the reservoir and readout (dashed arrows) were modified, through learning, to reproduce the behavior given by sequence of correct input/output examples. A contextual memory version of the model included a trained context neuron (in brown) that represented phase information (search or repeat). **B**. Time course of a modeled trial. A trial started with the activation of the lever and fixation point. Fixation point neuron deactivated concomitantly with targets appearance which was the GO signal for saccade to a target after a reaction time. Fixation of the target followed and was represented in the activation of one readout neuron among the four dedicated to target fixation. The lever input deactivation was the GO signal for arm touch that was represented in the activation of the readout neuron, after a reaction time, that represented the target chosen with the saccade in the second set of four readout neurons. Touch event occurred at the middle of arm choice and was the start of a 0.6 second delay to feedback. Feedback was simulated as the activation of the reward input for correct trials and the absence of activation in incorrect trials. A 2.15 seconds inter-trial interval started at the onset of feedback and ended at the onset of the next trial except for the last trial of problem (COR4: fourth correct trial) in which the inter-trial interval was extended to 4.25 seconds and the signal to change activated for 1.2 seconds. (Rwd: reward) **C**. Example of the network performing the task after learning to explore the targets with a circular search. Upper panel: A sequence of stimuli neurons activation. Middle panel: Activity of 4 example reservoir neurons. Lower panel: Readout of the network showing the successive choices of the model. The example shows the end of a repetition period where red target was rewarded. After signal to change input was activated (grey block), a new search for the rewarded target began with the exploration of blue target, then yellow and finally purple which was rewarded and then repeated.

In both versions, a recurrent network of firing rate neurons received task inputs and were fully connected to a readout layer, the output of the model (Fig 2A). Reservoir recurrent connections provide rich dynamics formed by nonlinear recombinations of inputs that evolve through time. Readout neurons activate to represent model's target choice, and feed back the choice through readout-reservoir connections. Reservoir-readout connections are the only modifiable connections of the model.

Several parameters define the reservoir. The principal necessary property for reservoirs is to have rich dynamics. The essential characteristics are to have a sufficient number of non-linear neurons that are sparsely and randomly connected. Fixed network parameters include networks size (1000 neurons), and standard values for input sparsity (10%), internal reservoir connection sparsity(10%), and spectral radios (0.9). The simulation time step is set at 25ms in order to give a reasonable granularity for comparison with the primate data. Fixed unit level parameters include the choice of the tanh non-linearity and the time constant or leak rate of the reservoir units. The tanh non-linearity is traditionally used in the echo state networks, but others can be used such as the Fermi sigmoid. However, reservoirs with Fermi neurons have been shown to have significantly smaller short-term memory [30]. The reservoir unit leak rate was optimized for performance, and was set at 375ms (15 network timesteps). Deviations from this value resulted in degraded performance.

### Single neuron dynamics

Neurons were simulated as leaky-integrator firing-rate units. Inputs were integrated over time with the following equation:

$$x(t+\Delta t)=\left(1-\frac{\Delta t}{\tau }\right)x(t)+\frac{\Delta t}{\tau }\left(W_{res}\cdot r(t)+W_{in}\cdot u(t)+W_{fb}\cdot z(t)\right)$$ (1)

where *x(t)* denotes the membrane potential vector of reservoir neurons, Δ*t* the time step (25 ms), *τ* the time constant of the leaky integration (375 ms or 15 time steps), *W*_*res*_ the reservoir internal-weight matrix, *r*(t) the firing rate vector of the reservoir neurons, *W*_in_ is the input weight matrix, *u(t)* the input neuron vector, *W*_*fb*_ the readout to reservoir weight matrix and *z(t)* the readout neuron vector. At each time step the firing rate *r(t)* of reservoir neurons was computed as the hyperbolic tangent of its membrane potential *x(t)* generating a nonlinearity in the dynamics of the neuron:

r(t)=tanh(x(t))

The readout unit activity was defined as the weighted sum of the reservoir-neuron firing rate:

$$z(t)=W_{out}r(t)$$

where *W*_*out*_ is the readout-weight matrix.

Experiments included a version of the model in which noise was added to the model to test its robustness and the effect of noise on performances and mixed selectivity. Noise with the same properties was injected during training and testing. Because a large proportion of noise in cortical populations has been found to be correlated among neurons [31], noise was simulated as a random Gaussian component added to the activity of input neurons:

$$u_{noisy}(t)=u(t)+N(0,\sigma )$$

where *N*(0,σ) is a vector the size of *u*(*t*) of pseudo randomly generated values following a Gaussian distribution of mean 0 and standard deviation *σ*. To assess the effect of noise injection into the model, values of *σ* ranging from 0 to 7 in increments of 0.5 were each used in 30 simulation instances.

### Neural network architecture

We implemented an RC model where a reservoir of 1000 recurrently connected neurons was fully connected to a readout layer. Learning took place only between the reservoir neurons and the readout units, at the level of the readout weights. Weights between reservoir neurons (internal weights) and between input and reservoir neurons (input weights) were stochastically generated and fixed. Input weights were generated with a uniform distribution in the interval [–1, 1] with a 0.1 probability of connection. Internal weights followed a Gaussian distribution (μ = 0, σ = 1) with a 0.1 probability of connection between each pair of neurons. These were scaled so that the largest absolute eigen-value of the weight matrix—commonly referred to as the spectral radius—was equal to 0.9. This ensured a dynamical regime allowing for sustained activity in the recurrent network without saturation. Activity in the network thus developed and integrated successive stimuli inputs so that activity at each time point represented the combination of previous and current inputs (reservoir computing principle).

Input neurons represented the major external features of the task (Fig 2A). They included 5 inputs, each represented by one neuron: the fixation point, the lever, the targets, the reward and the signal to change. Each of these neurons had a 0.1 chance of connecting with each reservoir neuron. Weights were generated following a uniform distribution in the interval [–1, 1]. The model generated outputs corresponding to oculomotor saccades and arm touches to the spatial targets corresponding to the monkeys' behavioral output and time course of the task events. A first set of 4 readout neurons represented the four possible target choices for eye saccades and a second set of 4 readout neurons represented arm touches. The highest activated neuron for each of the two sets represented the model's choice and both neurons were required to represent the same target in each trial. In the contextual memory version of the model, an additional readout neuron was trained to represent the phase (search vs repetition). In both versions, all reservoir neurons were connected to the readout neurons and constituted the only modifiable connections of the network. The readout neurons were connected back to the reservoir neurons to feed the choice information back to the recurrent neurons with a 0.1 chance of connection. These connections were generated prior to learning and remained fixed for the duration of the experiment. Connection weights were drawn from a uniform distribution between -1 and 1 for the choice outputs and for the contextual readout neuron in the contextual memory version of the model.

### Simulation protocol

We trained the model to learn a task that reproduces the major features of the actual task performed by monkeys (Fig 2B). Timing of these elements closely matched the actual monkey task in order to compare evolution of activity in dACC and reservoir neurons. Fixation point and lever were each simulated as the activation of their corresponding input neurons. They provided GO signals as they switched off for saccade to and fixation of a target, and for touching this target respectively. The readout neurons corresponding to these choices were trained to activate at their respective GO signal after a reaction time of 250 ms and deactivated after a 250 ms reaction time following touch. Following the fixation point, an input neuron represented the presence of the targets on screen and is deactivated after touch. Activation of the arm touch neuron started before the actual touch event to allow the neuron representing arm choice to reach full activation before switching off the targets input and to simulate the preparation and movement itself. Feedback was simulated with a reward input neuron that activated when a choice was correct. At the end of a problem, a fifth input neuron was activated to represent the signal to change indicating the start of a new problem. In the contextual memory version, the context neuron representing the phase was trained to activate when the signal to change input neuron was being activated, and to remain active for the duration of the search phase, until the first reward.

Each trial lasted 5550 ms (222 time steps), except for the last correct trial (COR4) that ended with the presentation of the signal to change and lasted 8050 ms (322 time steps). The task was taught to the reservoir with supervised learning using a matched set of <stimuli, desired output> pairs made of 600 problems. Readout neurons were trained to represent choice by activating to value 1 at periods of choice while remaining silent the rest of the time, thus acting like binary neurons. The training procedure employed a slightly modified version of the FORCE learning method developed by Sussillo and Abbott [20]. With the FORCE method, learning of connection weights between reservoir and readout neurons is based on an on-line process of weight adjustment that allows for sampling of the readout error by the system. Weights are corrected so that a small fraction of the readout error is fed back to the reservoir. Readout weights are successively modified to produce the target output while sampling deviations in the reservoir activity that result from readout feedback with a slight discrepancy between actual and desired output. Hence, the system learns to produce a stable readout even in the face of readout errors that are propagated to the reservoir. We used the recursive least-squares algorithm in combination with the FORCE learning principle to modify readout weights, as described in Sussillo and Abbott [20]:

$$W_{o}{{}_{u}}_{t}(t)=W_{o}{{}_{u}}_{t}(t-\Delta t)-\frac{e(t)P(t)r(t)}{1+r^{T}P(t)r(t)}$$

Where *e*(*t*) is the error before weights are modified and is defined as the difference between actual and desired output. The error of readout neuron *i* is defined as follows:

$$e(t)=W_{out}^{T}{}_{i}(t-\Delta t)r(t)-d_{i}(t)$$

where *W*_*out*_ is the weight vector between the reservoir neurons and the readout neurons and *d*_*i*_*(t)* is the desired output. *P(t)* can be assimilated to the matrix of all learning rates for each pair of reservoir and readout neurons and is modified as follows:

$$P(t)=P(t-\Delta t)-\frac{P(t-\Delta t)r(t)r^{T}(t)P(t-\Delta t)}{1+r^{T}(t)P(t-\Delta t)r(t)}\text{ with }P(0)=I$$

where *I* is the identity matrix.

To allow for better convergence of the weights, we modified the feedback from the readout to the reservoir generated with the original FORCE learning method. We blended the actual output, produced after weight modification according to the FORCE principle, with a clamped feedback i.e. a delayed version of the desired output. The proportion of clamped feedback and actual output varied smoothly and steadily during training, starting with only clamped feedback and ending with actual output. The signal *f(t)* was fed back to the reservoir and replaced *z(t)* in eq (1):

$$f(t)=\frac{t}{L}z(t)+\frac{L-t}{L}c(t)$$

where *L* is the full duration of training (entire 600 problem block) and *c(t)* the clamped feedback that is a 325 ms (13 time steps, determined through optimization) delayed version of the desired output. This delay greatly improved learning in our experiment. With a delayed desired output as clamped feedback, readout neurons had to learn to activate at the onset of fixation and arm choice without the correct and expected readout activity that would have been fed back to the reservoir with FORCE-learning fast adaptation of the weights. Likewise, when readout neurons should deactivate at the end of fixation and arm choice, the reservoir neurons still received the clamped activity resembling a readout that was not deactivated. Similar to the FORCE learning principle, this method allowed the learning algorithm to sample a higher number of time steps with discrepancies between actual and desired readout around the activation and deactivation of the readout neurons.

### Testing procedure

In order to assess the trained model's behavioral performance, a sequence of 200 problems was provided as input to the reservoir and the output choices were evaluated. The maximally activated neurons for saccade and hand choices had to match, and thus represented the model's choice. Trials where saccade and hand choices did not match were counted as errors. Performance was assessed on the basis of three rules: (1) do not repeat an unrewarded target choice; (2) repeat rewarded target choice once found; (3) while searching for the rewarded target, do not choose the target rewarded on the previous problem. Performance of the model was measured according to these rules. Trials that did not respect one of the three rules counted as an error. Error rate was defined as the number of trials that did not respect the rules over the total number of trials. In order to balance the length of the search period, the number of search trials was generated for each problem in advance. Thus, no target was predefined as rewarded, rather, after a predefined number of search trials (from 1 to 3), the reward was given and the behavioral output of the model was assessed according to the above described rules (for a similar method used to test human subjects, see Amiez, Sallet, Procyk, & Petrides [32]).

We are interested in the capacity of the model to perform the problem solving task. Previous detailed analyses of monkey behavior in this task have shown that the animals produced planned and structured search behaviors [23]. Rather than trying to reproduce trial-by-trial behavior of the monkeys, we trained the model on examples that followed the above rules, and then tested its performance and analyzed its activity. We generated training data based on three different search behaviors, among which two were structured. All three search behaviors used to train the model complied with the above mentioned rules. A fourth training set was created from data from one of the monkeys trained on this task [21]. We thus tested four training schedules: First, using a random search where the targets were explored in a different order at each problem. Second, using an ordered search where targets were explored following the same target sequence at each problem while avoiding the previously rewarded target in the sequence. In other words, the search always started with the same target, except if it was rewarded on the previous problem, and followed the same sequence, again, avoiding the target rewarded on the previous problem. Third, using a circular search where targets were explored in infinite repeating circle. As an example, let's define the repeating sequence upper-left (UL), upper-right (UR), lower-right (LR), lower-left (LL), UL, UR, LR and so on. If for a given problem, the rewarded target is UR, the search of the next problem will start with the next target in the sequence, namely, LR and continue with LL and UL until it finds the rewarded target. Fourth, the model was trained with the search behavior from monkey 1 who best solved the task. In order for the model to effectively learn the task, error trials from the monkey were removed from the behavior fed to the network. Khamassi et al. [23] provide a detailed description of the monkey's behavior with reinforcement learning models.

Reservoir neuron analyses reported here are based on the activity of networks that learned to explore targets with the circular search. Results did not differ when the model was trained with the ordered search. Fig 2C illustrates the activity of reservoir and readout neurons corresponding to a sequence of inputs once the task has been taught with a circular search.

### Neural data

Quilodran et al. recorded 546 neurons in the dorsal bank of the cingulate sulcus of two rhesus monkeys and analyzed them along with local field potential for their correlation with the behavioral shift [21]. The present article reports on a new and separate reanalysis of this dataset to support findings obtained with modeling. All reanalyzes of these data were based on firing rate estimates of the recorded neurons. Subsets of this pool of neurons were selected depending on the requirements of the analyses. The number of neurons per analysis is specified in each case in the related method description.

### Model and monkey single unit analysis

Mixed-selectivity analysis was performed by using the same methods for both reservoir neurons from the model (neurons from the recurrent network) and dACC neurons. The analysis focused on specific 500 ms trial epochs. Epochs used were: early fixation (0–500 ms from fixation onset), late fixation (-500–0 ms to targets appearance), before touch (-500–0 ms to target touch), before feedback (-500–0 ms to feedback) and after feedback (0–500 ms from feedback) (Fig 2B). Firing rates of reservoir neurons were averaged within these periods, thus obtaining a single firing rate value for each epoch. Average activity of dACC neurons for each epoch of each trial was estimated as the number of spikes within these epochs. Epoch, along with phase (search vs. repetition) and choice (UL, UR, LR, LL) constitute the three factors used in single neuron analysis with 5 (epoch), 2 (phase), and 4 (choice) possible levels respectively (40 conditions total). The dACC neuron pool for the mixed selectivity analysis was a subset of 85/546 dACC neurons selected for having at least 15 trials per condition. All reservoir neurons were included in the analysis. A three-way ANOVA was conducted on the activity of each neuron with factors Epoch x Phase x Choice. A neuron was considered significant for a factor or an interaction between factors if its p-value was inferior to 0.05 (corrected for multiple comparisons with false discovery rate across all neurons). Interaction effects between phase and choice are considered here as an indicator of mixed selectivity which is defined by the interaction of these variables in their contribution to the firing rate of a single neuron. Thus in this present study we use the term “mixed selectivity” to refer exclusively to its non-linear component. Moreover, we introduce the terminology “dynamic mixed selectivity” to refer to mixed selectivity patterns that interact with epoch and correspond in our experiment to the interaction between epoch, phase and choice variables in the ANOVA analyses.

In the monkey, responses specific to the first correct choice (COR1) were considered important as they mark the transition from search to repetition [21]. Thus, reservoir neurons of the model were also analyzed for their response to the first correct choice in a problem to compare with results obtained in Quilodran et al. [21]. For that purpose, firing rate activities of single reservoir neurons were averaged over the time window 300 ms to 800 ms after feedback onset and then pooled in incorrect (INC), first correct (COR1) and correct (COR) trials. Pairwise t-test with false discovery rate correction over all tests was used to quantify the number of reservoir neurons that fired significantly more in COR1 trials than in INC and COR trials (pooling tests of all neurons and all simulations, and with a threshold p-value of 0.05).

To demonstrate the presence of the COR1 information in the activity of the reservoir layer in each simulation, we trained an additional readout neuron to activate specifically for the first reward (reward during the COR1 trial) in a problem with the same method used to train other readout neurons. The successful learning of the COR1 readout neuron was assessed over 30 simulations with different pseudo-randomly generated weights according to the parameter values defined above.

### Model and monkey population analysis

Population analyses were performed on neural activity from full trials. Firing rate of each dACC neuron was first estimated with a Gaussian kernel (standard deviation = 100 ms) convolved through time every millisecond, eliciting a firing rate estimate at each millisecond. Activities were time normalized to accommodate for trial-time variations with the following method. The average duration of periods between key events of the task was calculated and allowed us to determine the number of time bins of a specified size (see below) within each period. The activity of each neuron was then divided in the number of time bins. For each neuron, estimated firing rate within these time bins was averaged to elicit a single average firing rate value per time bin. The events used to normalize time were: lever touch, targets appearance, target touch, feedback, next-trial lever touch.

We computed an autocorrelation on population activity of reservoir and dACC neurons to assess the dynamic nature of each of these populations. All reservoir neurons were included in the analysis. For dACC data, a subset of 290/546 neurons was selected for having at least 20 incorrect trials, 20 COR1 trials, 20 correct trials (excluding COR1 and COR4 trials) and 20 COR4 trials. The activity of each dACC neuron was time normalized using the above described method with 20 ms time bins. The activity of each neuron was averaged across all trials to derive population activity vectors for each of the time points composing a full trial (excluding signal to change period in COR4 trials). The resulting autocorrelation matrix is composed of all the Pearson correlation coefficient obtained from all possible vector-pair comparisons.

A decoding method was used to assess the capability of a linear readout to extract a continuous phase signal from the dynamic activity of the reservoir and dACC neurons. In the absence of a context neuron, a readout unit without feedback to the reservoir was trained to activate similarly to the phase context neuron, i.e. to start firing when the signal to change was given to the network, firing continuously during search phase and deactivate when the first reward was given during COR1 trials. With this method, phase information extraction had to rely only on the activity of the reservoir neurons. The result of this training produces a linear readout of the phase. Similarly, task phase was decoded from dACC population activity with time normalization (20ms bins) over all time bins of a trial after training a ridge regression on full trial activities. For training the decoder, the search trials included all INC trials, and the repeat phase was composed of COR2-3 trials. COR1 and COR4 trials lying at the transition between search and repeat were only used in testing. A subset of 290/546 neurons were selected for having at least 20 trials in each category (search/repeat). A linear readout model was derived from a linear regression between the full trial length (all time bins considered as an observation) and the desired output which was 1 for search trials and 0 for repeat trials. The output of the decoder was classified as correct if it was superior to 0.5 in search trials, and inferior to 0.5 in repeat trials. Ridge regression (Tikhonov regularization) was used to avoid overfitting of the linear readout/decoder. The ridge parameter was derived from a 10-fold cross-validation on the INC and COR2-3 trials: 2 test trials in each of the search and repeat categories, and 18 train trials in each category. The ridge parameter value obtained after optimization (10^−8^) was used for all the decoding analyses. To assess the separability of the search and repeat activities, the decoder was trained and tested on all INC / COR2-3 trials. For this first analysis, error rates were computed as the number of time bins (from all test trials combined) incorrectly classified over the total number of time bins. To demonstrate the generalization capabilities of the linear readout/decoder to new data, it was similarly trained on INC / COR2-3 trials and then tested on all the time bins of 20 COR1 and 20 COR4 trials. Permutations tests were performed to ensure the significance of the decoder: INC / COR2-3 labels were shuffled 10,000 times, which allowed to derive a 95% confidence interval for each time step. Every time step with a decoding accuracy equal or superior to the confidence interval was considered as significant.

A state space analysis was performed, allowing visualization of population activity trajectories, with a principal component analysis (PCA). Activities of each successive trial in a problem were averaged at the level of single neurons over each trial type: INC1 and INC2 were the first and second unrewarded search trials, COR1 the first rewarded trial, and COR2-4 the repeat trials with the presentation of the signal to change at the end of the COR4 trial. INC3 trials were ignored due to lack of trials in dACC data, but results with few trials show that INC3 trajectory was very close to INC2 trajectory and did not add relevant information to this analysis. A subset of 184 dACC neurons were selected for having at least 10 trials of each type. The activity of each dACC neuron was time normalized using the above described method with 100 ms time bins and then mean normalized over all bins for each trial type. Similarly, reservoir neuron activity was averaged over all trials of a trial type for each time point. PCA was performed on the data matrix where columns correspond to individual neurons and rows represent the concatenated time points of each trial type. Each cell in the matrix was the mean normalized average firing rate of one neuron at one time bin for one trial type. All reservoir neurons were included in this analysis.

## Results

### Model performance

The model learned to perform the task almost perfectly with all training protocols, except for the random search. As expected, it was impossible for the model to learn to perform the task with a random search (41.64% ± 10.91% of suboptimal choices over 30 simulations, 1000 reservoir neurons) (see Methods for full description of performance calculation). In the absence of a pattern in the trained search sequence, the model could not produce a coherent output. In contrast, the model performed the task almost perfectly with the circular search (0.11% ± 0.43% of suboptimal choices over 30 simulations, 1000 neurons) and the ordered search (5.92% ± 5.40% of suboptimal choices over 30 simulations, 1000 neurons). Interestingly, the model also learned successfully to perform the task when trained on a schedule derived from the performance of Monkey 1 after training (4.71% ± 4.35% of suboptimal choices over 30 simulations, 1000 neurons). As a comparison, the rate of suboptimal choices over all trials in monkeys assessed with the same method were 0.53% and 2.21% for each monkey, respectively. The observation that the reservoir model could learn the task, i.e. learning to repeat when it receives reward, and shift when it does not, represents a novel extension of the “cognitive” functions of reservoirs.

### Neural coding of task-related and behavioral variables

**Fig 3:**
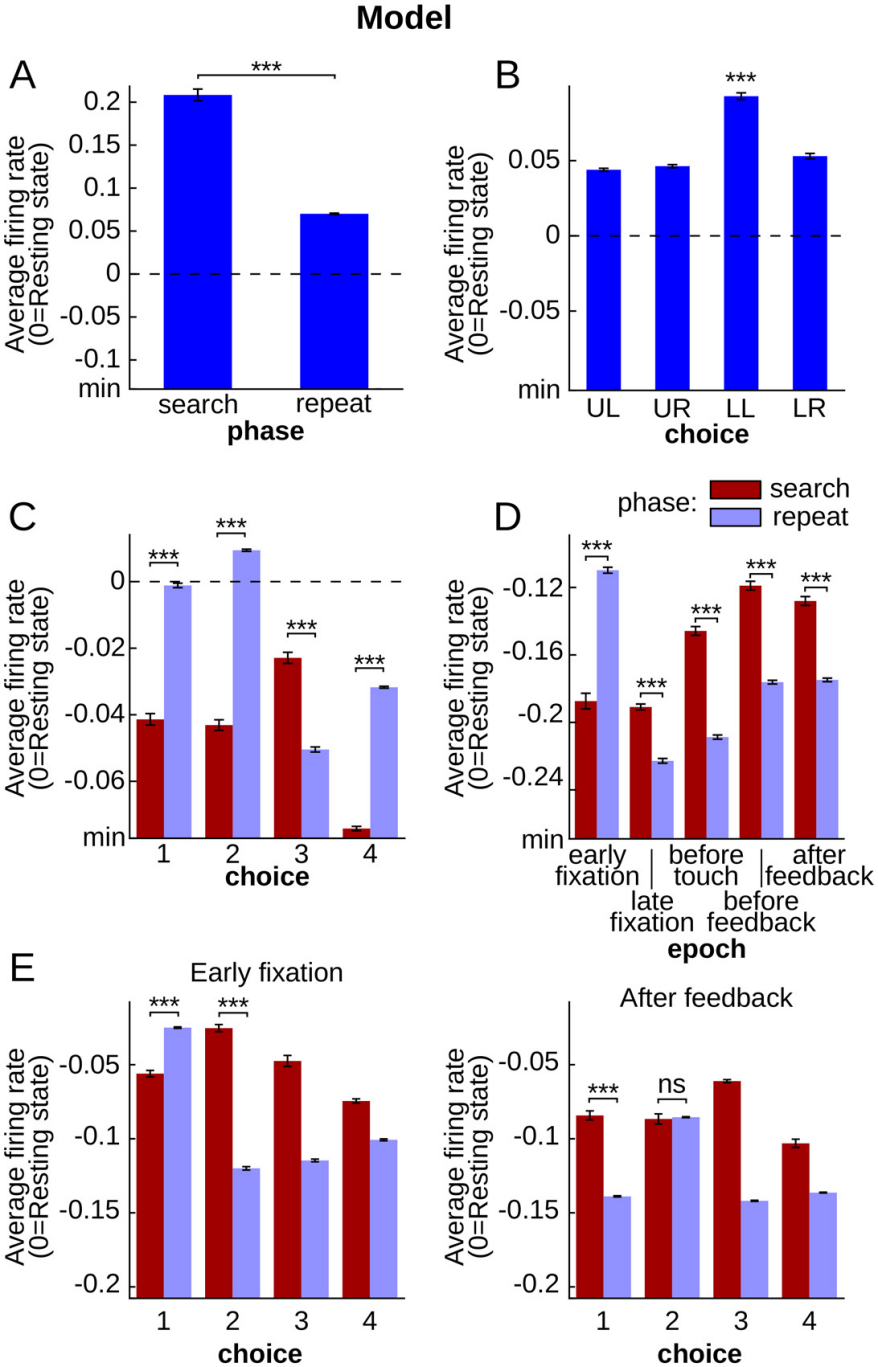
Activities of reservoir model neurons displayed single variable selectivity, mixed selectivity and dynamic mixed selectivity. Each histogram shows an example reservoir unit activity averaged over all trials of each given condition (error bars represent SEM). All statistical tests displayed on this figure correspond to two-sided pair-wise t-test with false discovery rate correction (p-value < 0.05). **A.** and **B.** represent the activity of two single units, respectively selective for phase at epoch late fixation and choice at epoch after feedback. **C.** Mixed selectivity pattern with significant phase-choice interaction (ANOVA, Phase x Choice interaction, p-value < 10^−15^) showing statistically higher activities for repeat phase when UL, UR and LR are chosen and the opposite pattern for LL target. **D.** Reservoir unit selectivity for phase depends on epoch (ANOVA, Epoch x Phase interaction, p-value < 10^−15^). **E.** Reservoir unit activity for all conditions of phase and choice at epochs early fixation and after feedback showing two different patterns of mixed selectivity (ANOVA, Epoch x Phase x Choice interaction, p-value < 10^−15^). Reservoir activity for phase depends on choice (left), and this dependence varies depending on epoch (right).

Because the model successfully learned to perform the task, it is of interest to examine the neural coding of behavior within the recurrent network, and compare it with that in the primate cortex. We examined the coding of two pertinent variables in this task: the target choice, and an internal variable, corresponding to the phase within a problem (search or repetition). To explore the variance in reservoir activity explained by these two variables, we systematically tested each neuron (total of 1000 neurons) in 30 independent simulations with the circular search using ANOVA, a commonly used parametric test in single-unit electrophysiology experiments [33, 34]. Tests were performed at 5 different epochs: early fixation, late fixation, before touch, before feedback and after feedback (Fig 2B). Fig 3A and 3B illustrate two typical examples of reservoir neurons that display activity profiles that code for phase, and choice, respectively. The presence of such neurons was tested by ANOVA. The majority of reservoir single units displayed significant main effects for choice and phase (three-way ANOVA, Epoch x Phase x Choice; mean ± std: Phase 97.5 ± 0.6% of a total of 1000 neurons; Choice 99.8 ± 0.2%, see Fig 3A and 3B for two example units). These results did not differ with the ordered search training.

**Fig 4:**
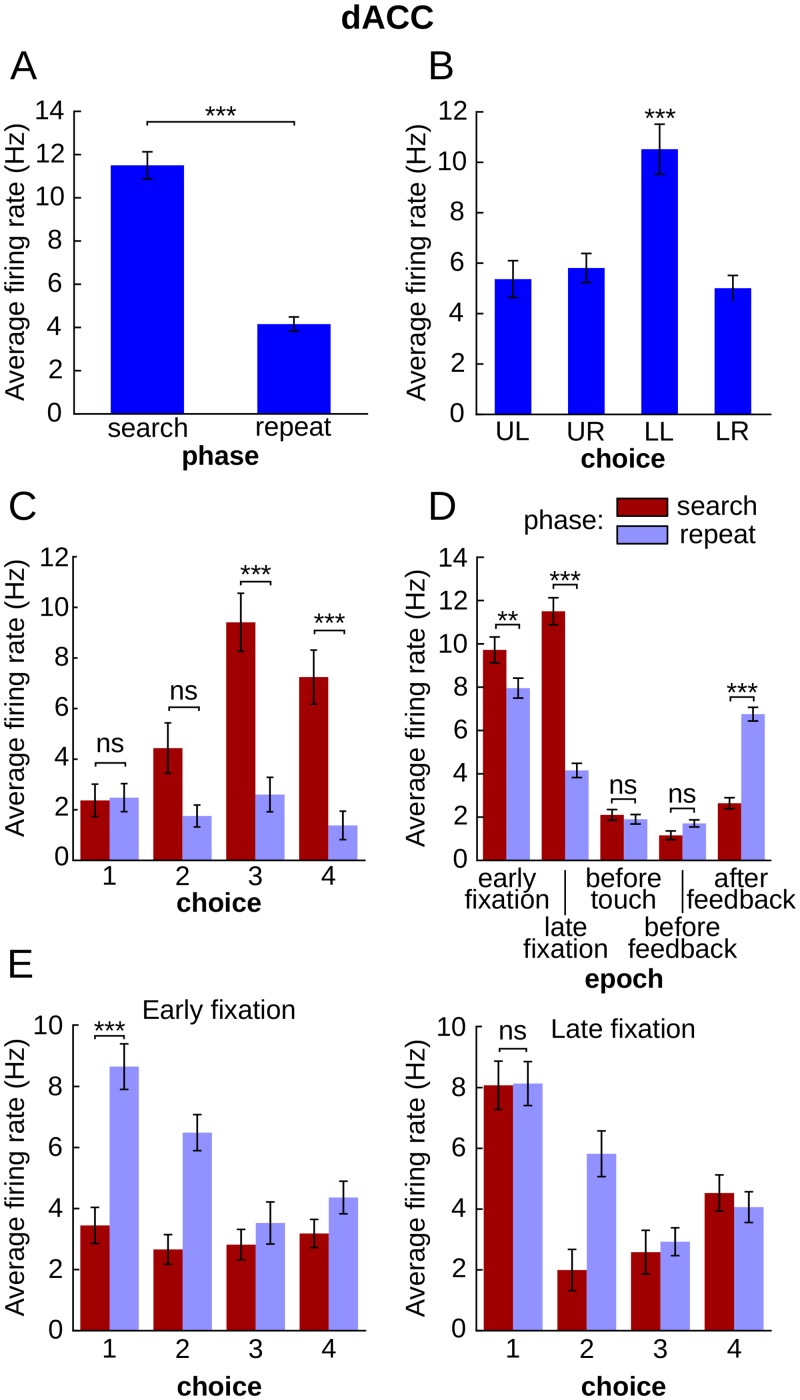
Similar to Fig 3 for dACC example neurons. **A.** and **B.**, single task variable selectivity. **C.** A dACC neuron activity significant for phase choice interaction (ANOVA, Phase x Choice interaction, p-value < 10^−5^) that is selective for phase only when lower left and lower right targets are chosen. **D.** dACC unit selectivity for phase depends on epoch (ANOVA, Epoch x Phase interaction, p-value < 10^−15^). **E.** Pattern of phase-choice mixed selectivity that changed depending on the epoch (ANOVA, Epoch x Phase x Choice interaction, p-value < 10^−3^).

Focusing on pre and post feedback epochs, Quilodran et al. [21] showed that some dACC neurons respond differentially depending on the phase of a problem. For the purpose of comparison with the model, we generalized this approach by assessing the difference in activity of the 85 dACC neurons that had at least 15 trials per condition with the same method used for the model: 57 neurons displayed a significant effect for phase (67.1%) and 49 a main effect for choice (57.6%) (Fig 4A and 4B).

Thus, single units within the recurrent reservoir network and the dACC encoded task related variables including target choice, and task phase, as revealed by significant main effects in ANOVA. Neither the target choice, nor the phase could be directly derived from the current inputs, whether they be external inputs (reward for phase variable) or feedback of responses (choice feedback for choice variable). Rather, they depended on the history of previous inputs and responses. This selectivity for previous inputs and states in reservoir activity is due to the recurrent dynamics and is part of the defining properties of reservoir computing [8–10]. While such task related activity invites a straightforward explanation of cortical function, as noted above, recent research suggests that cortex does not uniquely rely on such simple coding schemes, and also displays more complex mixtures of selectivity to task related variables [7].

### Mixed selectivity

As just seen, analysis of neural activity in terms of single task variables can provide an explanation of neural coding that appears simple. However, the activity of PFC neurons has often been described as complex, reflecting different combinations of task-related variables [3–6]. This phenomenon has been the focus of several recent studies which revealed its importance for cognitive tasks, and may underlie the capacity of the cortex to represent any contingency explained by a combination of task variables [7]. Fig 3C and 3D illustrate these mixed selectivity effects in the reservoir: coding of phase was dependent on choice, and epoch, respectively. The presence of such neurons was tested by ANOVA. The analysis of Choice x Phase interactions can reveal whether reservoir neurons display mixed-selectivity properties (three-way Epoch x Phase x Choice ANOVA, Choice x Phase interaction, p-value < 0.05; see Methods). Nearly all reservoir neurons showed mixed selectivity effects (99.9 ± 0.1% out of 1000 neurons, over 30 simulations) as revealed by the Choice x Phase interaction (Fig 3C).

While mixed selectivity has been described in PFC neurons, no study has yet to our knowledge systematically and specifically explored it in the dACC. We thus tested the prediction that this same mixed selectivity, as revealed by the Choice x Phase interaction, should be present in dACC neurons in animals trained to perform the same task as the reservoir model. Our reanalysis of the Quilodran et al. data by ANOVA indeed revealed that 28 out of 85 dACC neurons (32.9%) selected for the analysis displayed mixed selectivity (Fig 4C).

Rigotti et al. [2] showed that neurons of recurrent networks display complex recombination of current input that are similar to mixed-selectivity activities observed in PFC. These recombinations would allow external units connected to the recurrent network to detect the high dimensional combinations of multiple inputs relevant to the task to be learned. Recurrent networks of the reservoir type have the double property of recombining inputs and maintaining information about inputs across time [8–10, 25]. This is exemplified in the current experiment as the recombination of phase and choice information expressed in mixed selectivity that cannot be explained by a simple linear combination of the two variables contributions, and is dependent on the history of previous inputs.

### Dynamic mixed selectivity

Modulation of selectivity through time is a well described feature of PFC neuronal activity, whether the neurons show selectivity at a specific time in a trial or shift selectivity within a trial [33–35]. We will refer to this pattern of selectivity that changes over time as dynamic selectivity. Model reservoir neurons displayed clear dynamic selectivity, with nearly all the neurons showing modulation of selectivity across epochs for both phase and choice task variables as revealed by Epoch x Phase and Epoch x Choice interactions (mean ± std: 99.2 ± 0.4% for Epoch x Phase interaction and 99.4 ± 0.4% for Epoch x Choice interaction for 1000 reservoir neurons across 30 simulations, three-way ANOVA, Epoch x Phase x Choice, p-value < 0.05, Fig 3C and 3D). Single variables were encoded dynamically which suggests that mixed selectivity could be encoded in a dynamic fashion as well.

Pursuing this dynamic aspect, in the following, a pattern of mixed selectivity that changes over time is referred to as dynamic mixed selectivity. Fig 3E illustrate these dynamic mixed selectivity effects in the reservoir: the interaction between phase and choice is itself dependent on the task epoch. We consider dynamic mixed selectivity when there is a significant three-way interaction between task epoch, phase and choice with the ANOVA. Dynamic mixed selectivity was observed in the majority of model neurons (99.2 ± 0.6% of 1000 neurons, three-way ANOVA, interaction between Epoch x Phase x Choice, p-value < 0.05, Fig 3E).

In order to determine if this property of the reservoir was equally observed in the primate data we performed the three-way ANOVA and examined the two way and three way interactions. Dynamic selectivity was similarly found in the majority of dACC neurons (63 out of 85 neurons (74.1%) for Phase x Epoch interaction, and 49 out of 85 neurons (57.6%) for Choice x Epoch interaction, Fig 4C and 4D), as well as dynamic mixed selectivity, as revealed by the Epoch x Phase x Choice interaction (14 out of 85 neurons, 16.5%,Fig 4E). This prevalence of forms of dynamic selectivity suggests that the characterization of neurons as showing mixed selectivity features through the interactions of two variables excluding time can be extended to include the dynamical nature of these mixed-selectivity representations.

### Effect of noise on model unit selectivity and performance

**Fig 5:**
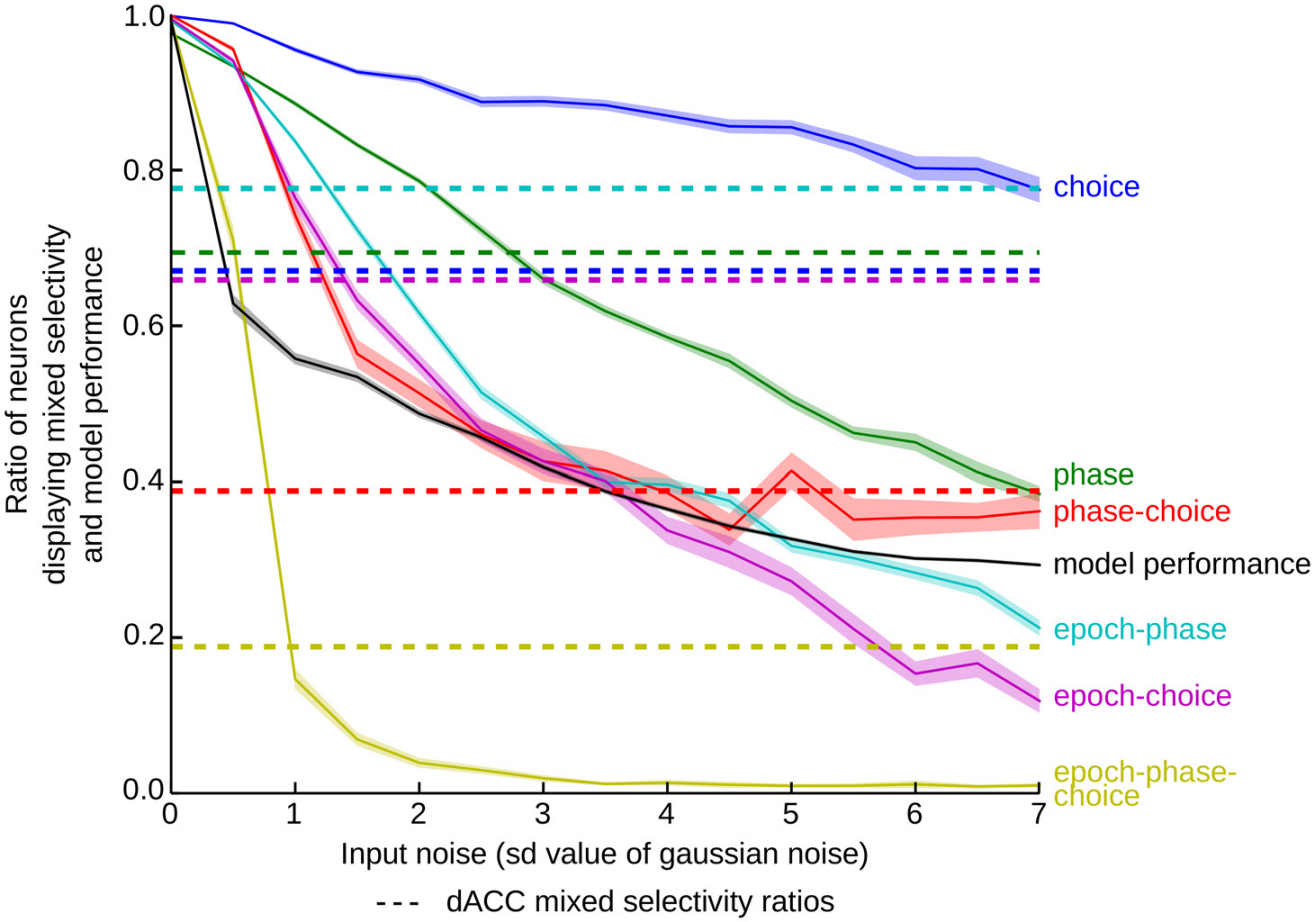
Mixed selectivity and performance of the model as a function of noise. Each curve represents the average ratio of model units that are significant for one of the tests. Tests include selectivity for factors Choice, Phase, and the interactions Phase-Choice, Epoch-Phase, Epoch-Choice, Epoch-Phase-Choice (3-way ANOVA, Epoch x Phase x Choice, shaded areas correspond to standard error). For comparison purposes, the dACC ratios are plotted as dashed lines with the same color code (see legend). On a similar scale is represented the performance of the model (black line) as the ratio of trials with a correct answer over the total number of trials. All selectivity ratios decreased with increasing noise, more complex selectivities decreasing faster. As dynamic mixed selectivity dropped with the slightest noise, so did the performance.

Most of the neurons in the model were significant for each test, while dACC neurons displayed high percentages of neurons selective for main effects of phase, choice and epoch, and then progressively reduced percentages for two- and three-way interactions (Table 1). This suggests that these higher order mixed selectivity properties are more fragile, and might be potentially susceptible to the addition of noise to the system. Indeed, the simulations were performed in the ideal noiseless condition. Injecting noise into the model let us explore the robustness of all types of selectivity (simple, mixed and dynamic mixed selectivity). We used progressively increasing Gaussian noise that was added to the input fed to the network during training and testing (see Methods for details). Increasing noise elicited a decrease in the scores of all types of selectivity (Fig 5). Interestingly, the more complex was the selectivity, the faster the number of unit displaying this selectivity dropped. The single task variable selectivities decreased more progressively than mixed selectivities, and the dynamic mixed selectivity was the most sensitive to noise. Interestingly, the model performance decreased directly with the mixed selectivity.

**Table 1:** Results of a three-way ANOVA (Epoch x Phase x Choice, FDR correction on all tests, all neurons, all simulations, p-value < 0.05) on reservoir (average ± std out of 1000 neurons over 30 simulations with 1 SD Gaussian noise) and dACC neurons showing the number of neurons significantly modulated by the factors shown on the first column.

	Model—1.0 noise SD	dACC	
	Average # of significant neurons	Significant neurons	
Phase	885.4 ± 19.2	57	67.1%
Choice	954.5 ± 18.2	49	57.6%
Epoch	999.8 ± 0.5	83	897.6%
Epoch * Phase	836.9 ± 25.4	63	74.1%
Epoch * Choice	764.2 ± 66.1	49	57.6%
Phase * Choice	742.0 ± 69.6	28	32.9%
Epoch * Phase * Choice	146.6 ± 71.0	14	16.5%

Selectivity ratios similar to that of the dACC population were obtained in the model with varying standard-deviation of noise, the tendency being that the more complex selectivities require less noise to attain the corresponding ratios of the dACC. We calculated the Pearson r correlation between number of significant neurons for each test the dACC, and the model with different levels of noise. As illustrated in Table 2. With noise from 1 to 4.5 SD, the p-value of the correlation is below 0.05 (see Table 1 for a comparison between dACC and the model with a 1 SD Gaussian noise). However, in this range of noise the model performed poorly. Interestingly, the performances dropped with slight noise, as was the case with the dynamic mixed selectivity. Although this result cannot be accepted as a causal relation between dynamic mixed selectivity and performance, it is reminiscent of the correlation observed by Rigotti et al. (2013) between non-linear mixed selectivity and monkey performance.

**Table 2:** Correlation between significant number of neurons for each test in the 3-way ANOVA between dACC neurons and model neurons with different levels of noise.

Noise SD	0	0.500	1.000	1.500	2.000	2.500	3.000	3.500	4.000	4.500	5.000	5.500	6.000	6.500	7.000
Pearson r	-0.01	0.726	0.823	0.866	0.846	0.825	0.804	0.777	0.775	0.776	0.707	0.710	0.691	0.678	0.640
P-value	0.988	0.065	0.023	0.012	0.017	0.022	0.029	0.040	0.041	0.040	0.075	0.074	0.086	0.094	0.122

Reservoir networks are by nature dynamic, and representations within single reservoir neurons are themselves highly dynamic. Likewise, it was demonstrated that the presence of mixed selectivity in the activity of single PFC neurons is important for behavior [7], and that this feature may be necessary to represent conjunction of variables that are not represented in neurons firing only for single variables [2]. Still, it is relatively non-intuitive to understand the role of dynamic mixed selectivity in single units for producing complex but stable behaviors, leading to questions concerning how any pertinent information can be extracted from such unstable coding.

The current analyses reveal (1) task related neural activity, (2) mixed selectivity, and (3) dynamic mixed selectivity. The question remains, what is the underlying meaning or content of these representations? In the past we have seen “meaningful” forms of mixed selectivity, as in the combined retino-topic and sequence rank effects in PFC during sequencing tasks [4]. What about in the current case? Are there forms of mixed selectivity that can be observed to be meaningful in this task? Can we extract this information in a meaningful way? Can we visualize these representations? These questions will be addressed in the following sections.

### Task-relevant mixed selectivity

**Fig 6:**
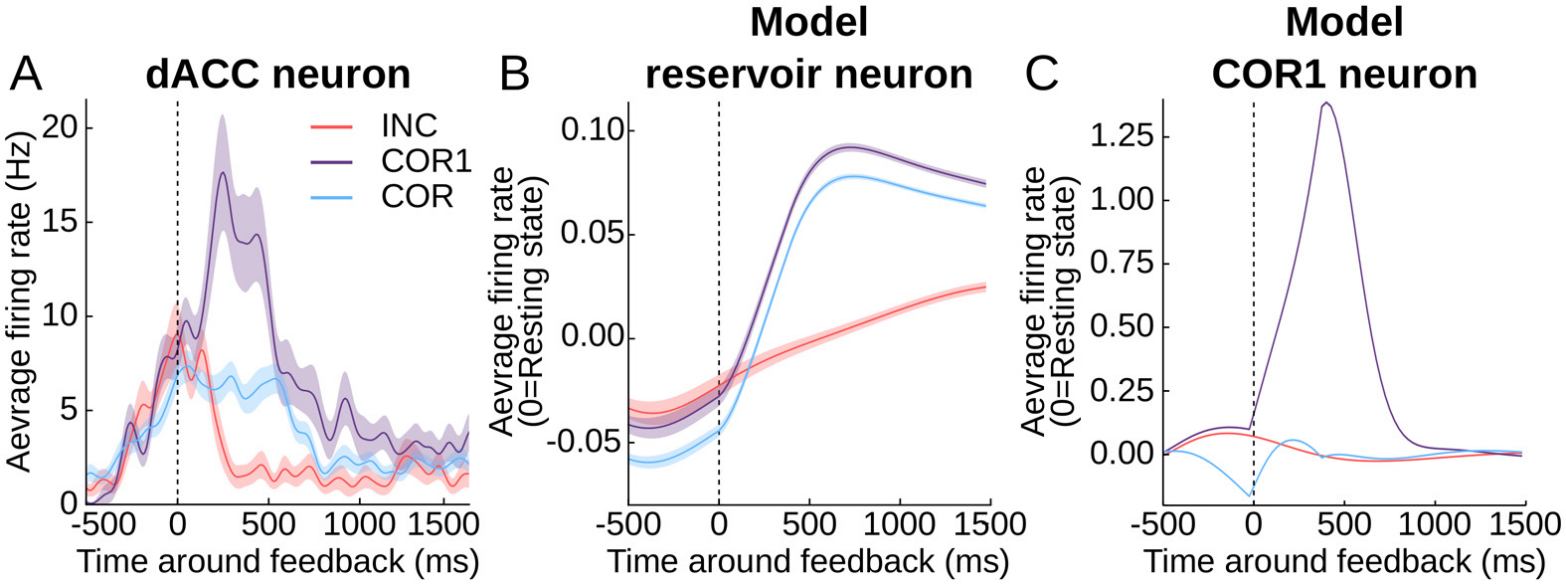
Example activities of reservoir and dACC neurons showing higher activity for feedback in COR1 trials. Single neurons activities are averaged over incorrect (INC), first correct (COR1) and other correct (COR) trials and centered on the feedback (shaded areas correspond to SEM). **A**. Average activity of a dACC neuron showing a strong increase in activity after feedback for COR1 trials only (pairwise t-test with false discovery rate correction, COR1 > COR corrected p-value < 10^−3^, COR1 > INC corrected p-value < 10^−5^, Gaussian smoothing of firing rate with 35 ms standard deviation). **B**. Reservoir neuron from the model showing a significantly higher activity after feedback for COR1 trials (pairwise t-test with false discovery rate correction, COR1 > COR corrected p-value < 10^−6^, COR1 > INC corrected p-value < 10^−15^). **C**. Model readout neuron trained to activate specifically for the first reward, demonstrating the presence of the COR1 information in the reservoir.

We have seen that certain neurons encode uninterpretable forms of mixed selectivity (e.g. Figs 3 and 4C and 4D). Are there forms of dynamic mixed selectivity that are more meaningful? In the problem solving task the first reward is the key transient signal to stop exploring and concentrate on the rewarded target, i.e. to initiate the repeat phase. Quilodran and colleagues found that this transition signal is represented in the activity of a sub-population of dACC neurons. These feedback-related neurons activated only after the feedback of the first rewarded trial (COR1, see Fig 6A), and thus represented the conjunction of reward and search phase. Their activity was significantly different from incorrect (INC) and correct trials in repeat phase (COR) (12% and 24%, of 146 and 88 feedback related neurons in first and second monkey respectively, see Quilodran et al., [21]). This is an example of mixed selectivity that is highly relevant for the task, as a signal to shift from the search phase to the repetition phase, each of which has specific and distinct behavioral requirements.

In order to determine if neurons in the reservoir displayed this task-relevant mixed-selectivity COR1 response, we compared the average firing rate during the post feedback period (300ms to 800ms after the feedback) and found that 1.1% ± 1.1% (mean ± std, 30 simulations, example in Fig 6B) of the neurons had a significantly higher firing rate for the first rewarded trial than for the unrewarded and other rewarded trials (one-sided pairwise t-test with Bonferroni correction for each neuron, p-value < 0.05). Correcting p-values with FDR over all neurons of all simulation elicits 0.24% ± 0.3% significant COR1 neurons. Following correct statistical procedure, it seems that the number of COR1 neuron in the reservoir is 0, and all observed COR1 neurons are due to chance only. Furthermore, Fig 6B clearly shows how the difference in activity between correct and COR1 trials is slight. However, each of the 30 simulations without exception produced COR1 neurons (minimum 2—maximum 65). Indeed, one of the principles of a recurrent network with random connections is that “by chance” some neurons will show a pattern of activity corresponding to a given combination of inputs and/or internal states. While the number of neurons encoding the COR1 information in the reservoir is not statistically significant, their consistent presence should allow for extraction and strengthening of the signal for more explicit processing. It is possible that the COR1 response in the monkey was increased by learning to allow the system to represent a task relevant information more explicitly.

To illustrate this point, we trained an additional readout neuron to activate specifically when the first reward of a problem was given to the reservoir. We reasoned that if the information is robustly present in the recurrent network, the training of a COR1 readout neuron should be straightforward. Indeed, each of the 30 instances of the model trained to produce a COR1 neuron were successful (Fig 6C illustrates the activity of the COR1 readout of an example simulation). Therefore, the COR1 information was present, distributed in the activity of the reservoir population.

### Full trial population dynamics

The single unit analyses revealed that mixed selectivity changes in time—that is, it is dynamic. To further demonstrate the dynamic nature of neural activity at the population level, we used an autocorrelation method on the reservoir neural population. By correlating successive population activity vectors, this method allowed us to reveal how fast the population activity is changing. The activity of each neuron at each time point within a full trial was averaged over all trials. Results of the autocorrelation are represented on a heat map in which every point represents a correlation between the population activity vectors at two different time points within a trial. The diagonal represents the correlation between one time point with itself and will necessarily elicit a correlation coefficient of 1. Slow variations in the activity pattern elicit extended correlation around the diagonal, while dynamic coding in the population elicits narrow bands along the diagonal line.

**Fig 7:**
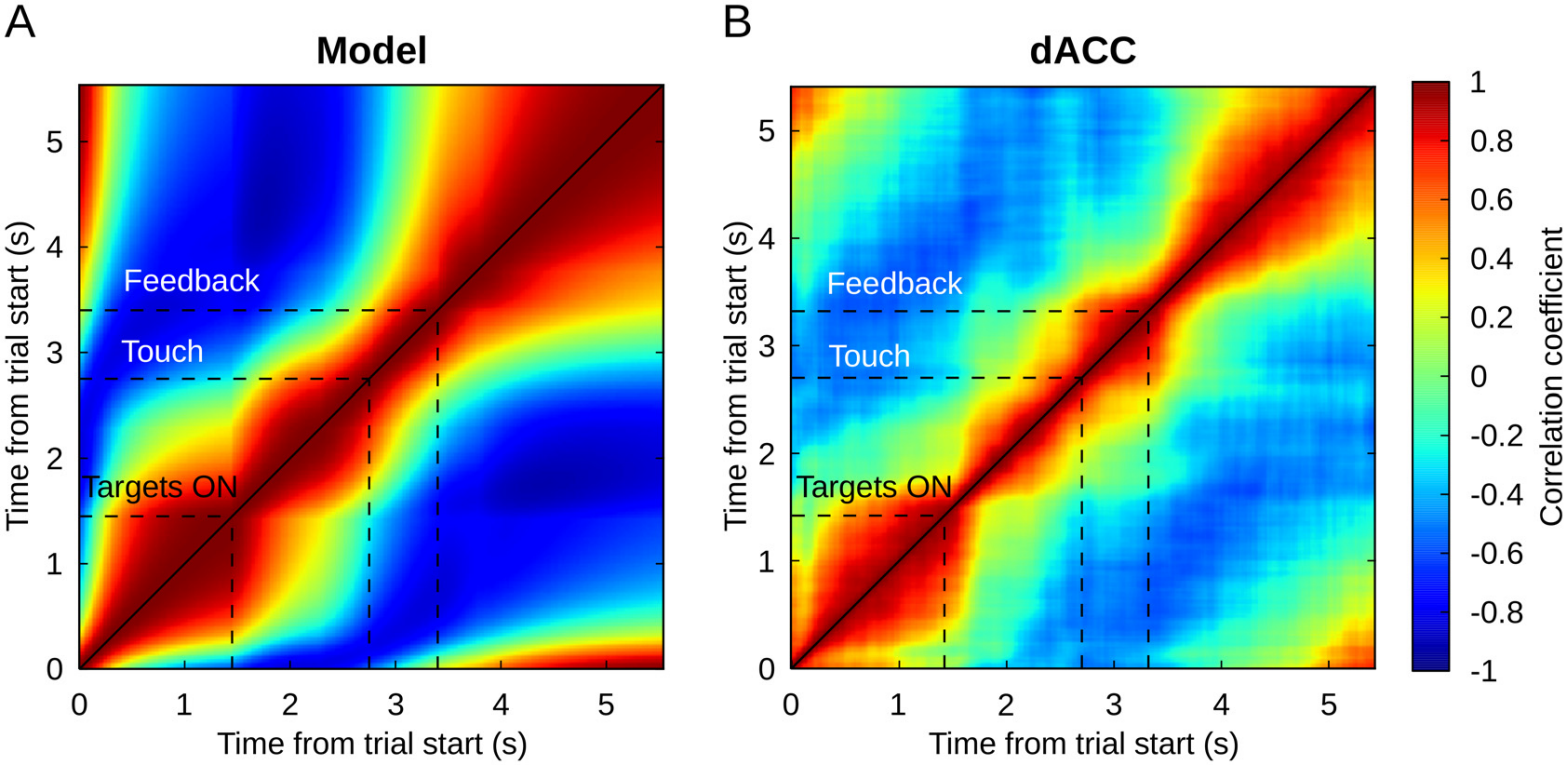
Autocorrelation of the model (A) and the (B) dACC population activities. Each point in this graph represents the correlation coefficient between the population activities at two different time points, except along the diagonal where each point is the correlation of the activity with itself. Both model and dACC populations show a dynamic pattern of activity that is closely following the sequence of the main events in the task.

Results show a band of positive correlations largely concentrated around the diagonal, which confirms the globally dynamic character of representations observed in single unit analyses (Fig 7A). As expected from a reservoir network exhibiting the echo state property, the shape of the correlation pattern follows the precise sequence of inputs fed to the network. The narrowest pattern along the diagonal is observed at the beginning of the trial (lower left corner) when both the lever and fixation point inputs were activated, triggering a strong change of activity in the population. Similarly, successive events of the task (indicated by dashed lines in Fig 7A) are associated with a following narrowing of the correlation pattern, while absence of inputs left the network in a more stable and persisting state. A more stable pattern of activity follows the feedback, corresponding to the inter-trial period when no inputs were active. Here in the absence of input the network activity begins to stabilize, but still retains sufficient coding of the phase so as to continue correctly in the next trial.

Applied to the dACC population activity, this method displays a strikingly similar correlation pattern: dynamic activity at trial start, after target appearance and after feedback and more stable activity between these events (Fig 7B). Event related narrowing patterns appeared slightly later in the case of the dACC, probably because the inputs associated with these events reached the dACC through other brain regions, whereas the reservoir network was directly connected to the inputs. A widening of the correlation pattern between touch and task feedback represents the sole main discrepancy between dACC and model results.

The similarity between model and dACC may also be explained by a similar time constant in the model and dACC. The time constant *τ* of the neurons in the model was first obtained through optimization, based on the performances of the model to perform the task. Interestingly, Murray et al. [36] recently found an empirical time constant of ACC neurons (between 257 ms and 340 ms) relatively close to our optimized value (375 ms). Among the prefrontal areas investigated in this study ACC has the highest intrinsic time constant.

Overall, on the period of a full trial, these results suggest that population activity patterns are quite dynamic in both dACC and model, suggesting a transient dynamic which is a characteristic of reservoir systems. For the model these transient patterns depend on the inherent network dynamics, and the externally imposed task schedule. The similarity with the dACC results suggest that the dynamic character of this area can be explained by the same mechanism.

### Dynamic mixed selectivity can generate stable output

We have shown that single unit (Figs 3E and 4E) and population activities (Fig 7) in the model and the monkey displayed complex and dynamic activities that are not easily interpretable as to their contribution to stable and controlled output. That is, the mapping of this complex time-varying activity onto coherent behavior is not immediately evident. However, both monkeys and the model were quite efficient at performing the task. This implies that in both, there was a mechanism that can extract coherent task-relevant representations from dynamic activity. Recent studies suggest that complex non-linear combinations of inputs create rich activities in recurrent networks from which relevant information is easily extracted using simple linear regression [7, 37]. In other words, RC networks expand the input space into a rich state space, now composed of spatial and temporal information. The role of learning is to find a linear readout that best separate states representing relevant information.

**Fig 8:**
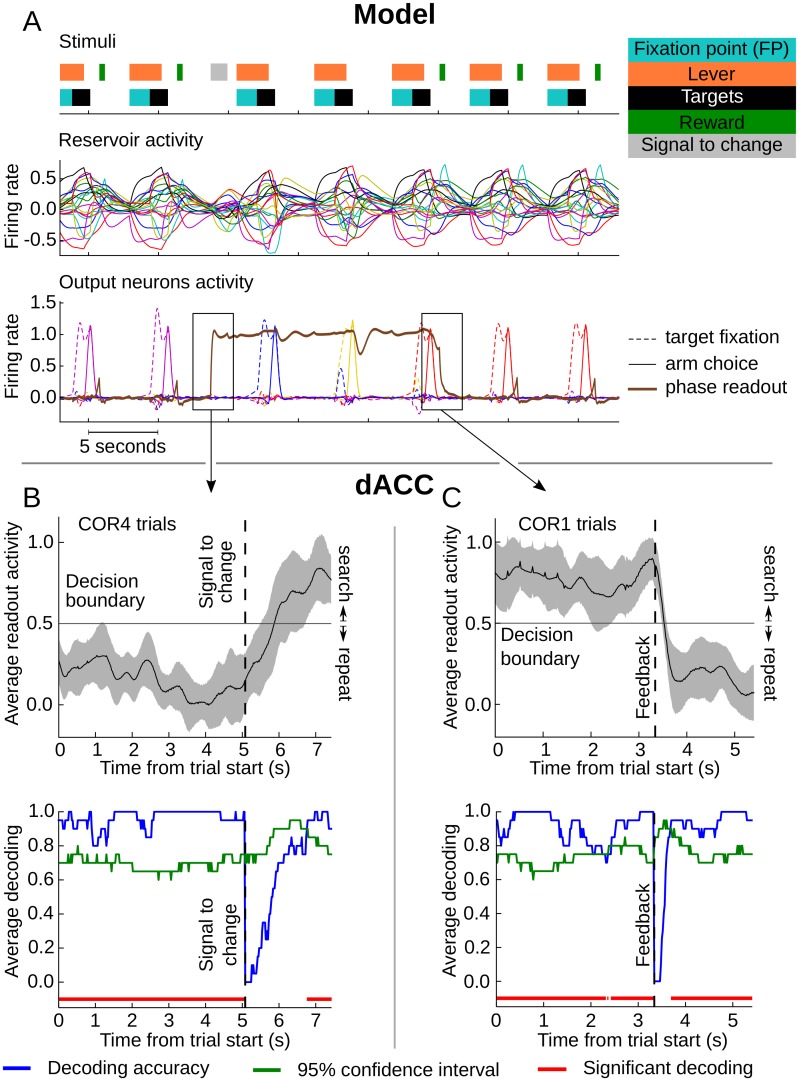
Continuous phase readout from reservoir and dACC activity. **A**. Example stimuli sequence, corresponding reservoir activity and model output during test showing the stability of phase readout. Upper panel shows a selection within the stimuli input sequence fed to the reservoir during test. Colored blocks represent activity of the reservoir input neurons simulating the different features of the task. Middle graph shows the activity of 20 randomly picked reservoir neurons illustrating the dynamic nature of activity in the reservoir. Lower graph shows the evolution of the readout neurons that represent target fixation (dashed lines) and hand touch (solid lines) along with the activity of a unit reading out the task phase (brown line). This special readout unit was not connected back to the reservoir and could not rely on the attractors created with a feedback connection. This portion of stimuli sequence starts with the last trial of a problem. After the signal to change is given to the model (grey block) the phase readout unit activates and fires steadily during the following three trials corresponding to the search phase showing the stable output that can be obtained from the dynamic reservoir activity. The third target exploration is rewarded (COR1 trial) and ends the search phase. At COR1 trial, when the reward neuron activates, the phase readout unit shuts off thereby signaling the start of the repeat phase. The rewarded target choice (red lines) is then repeated in subsequent trials. **B**. Upper graph illustrates the average readout output across all COR4 trials. Shaded area corresponds to the standard deviation. Correct readout is inferior to 0.5 for time bins before the signal to change, and the opposite for the following time bins. Lower graph is the average decoding accuracy and 95% confidence interval as computed from a 10,000 permutation test. **C**. Same as **B** for COR1 trials. Correct readout is superior to 0.5 for time bins before the first correct feedback, and the opposite for the following time bins. Transition from repetition to search was slower than the transition from search to repetition.

Here, we attempted to determine whether a linear readout could be used to generate meaningful stable outputs across time by separating relevant states of a dynamic and complex activity. For this purpose we trained a readout unit, with the method used for other readout units, to represent the task phase (i.e. whether the system is in search or repetition) without feeding it back to the reservoir. Thus, the readout unit had to extract phase from a reservoir population that did not receive this readout as a feedback. The learning procedure converged on weights that activated this special readout neuron throughout the search phase even though phase representation was dynamic. Fig 8A illustrates the activity of target choice readout neurons of the model, along with the new trained readout unit extracting phase after the task has been learned (results were replicated on 30 simulations). The output of the phase readout unit was steady during search phase and activated and deactivated sharply with key events (signal to change and first reward). This demonstrates that a stable, task relevant signal (here the state of whether the system is in search or repeat phase) can be read out from the complex dynamic mixed activity generated in a reservoir network.

A simple linear readout could extract the task phase steadily from the reservoir population activity that seemed dynamic. Can we demonstrate the same decoding for cortical neurons? Astrand et al. [38] recently showed that one of the best decoders for task related variables on a population of prefrontal neurons is a simple regularized linear-regression which elicits performance similar to those using complex machine learning methods such as Support Vector Machines, while being simpler and less expensive computationally. We thus set out to reproduce the continuous decoding results observed with reservoir neurons, now with dACC activity, by training a readout through ridge regression. The readout was trained and then tested on the activity of full search (INC) and repeat (COR2-3) trials to see if it could continuously decode task phase (290/546 neurons having at least 20 trials of each category, ridge parameter obtained through cross-validation, see Methods for details). This method allowed us to assess if a single readout, that can be considered as a downstream neuron connected to the dACC population, can continuously extract phase information from a dynamic population activity. On average, over all time bins of every search and repeat trials tested, the decoder made only 3.0% ± 2.1% of errors (mean ± std). We then tested this decoder, that was trained on all INC and COR2-3 trials, with data not used in training, from COR1 and COR4 trials. This allowed us to explore the transition between search and repetition (COR1), and between repetition and search (COR4) in decoding phase with the linear readout. For both COR1 and COR4, decoding accuracy was very high in the period preceding the transition. The representation of the new phase built up gradually after the transition event (signal to change for COR4 trials, feedback for COR1 trials). Considering the time bins with significant decoding, the search to repeat transition occurred considerably faster (~300 ms) than its repeat to search counterpart (~1.5 s). Overall, apart from the transition period, the decoder performed extremely well, and thus demonstrated the success of a simple linear readout to continuously extract phase information from a dACC population whose phase representation was globally dynamic. In addition, this suggests that the states representing phase within the population activity belong to well separated areas of the population activity state space.

### Effects of explicit contextual memory

So far we explored the coding schemes of a classic reservoir, and demonstrated that its recurrent property produced a rich dynamic activity composed of input recombinations, in accordance with the universal spatio-temporal representational power of reservoirs. However, we showed how the influence of past inputs on the activity of the reservoir is limited in time, which consequently limits the time aspect of the reservoir representational power. To circumvent this inherent limitation, several studies in the reservoir computing community introduced units in the reservoir to explicitly represent a contextual information over time that will thereby influence the processing in the rest of the reservoir [17, 19].

In this context, we observed that the reservoir required a high number of neurons (on the order of 1000) to perform the task optimally. Phase information in the network (i.e. whether the system is in search or repetition phase) was the consequence of the reward, yet this short reward input had a limited influence on the reservoir dynamics due to fading memory [39] as shown by the auto-correlation analysis. In the previous section we demonstrated that phase information could be steadily extracted from the activity of the reservoir. To explore the effect of making this phase information explicit to the reservoir, we trained one additional output neuron to represent phase explicitly and fed it back to the reservoir (explicit context version). For the current analysis, this readout neuron was connected to the reservoir neurons by modifiable connections, and fed back its activity to the network as well (see Fig 2A). Like the phase readout, it was trained to activate steadily only during the search phase, hence mimicking the tendency of numerous dACC neurons to display higher activity in search versus repeat phase [21]. We refer to this as a “context” neuron as it encoded the phase, which provided a form of behavioral context. After learning, the context neuron became activated following the signal to change input at the end of a problem, and fired steadily until the first reward. Note that the activation of the phase neuron was learned and was produced solely through learning of the readout connections to this phase neuron, just like the other readout neurons. The only difference with respect to other output neurons is that rather than coding an actual behavioral response, it coded an internal state variable, the phase (search or repetition). Also, a major difference with the phase readout of the previous section is that because the contextual neuron fed its activity back into the reservoir, the contextual neuron operated in a loop in which it both influenced and was influenced by the activity of the reservoir.

**Fig 9:**
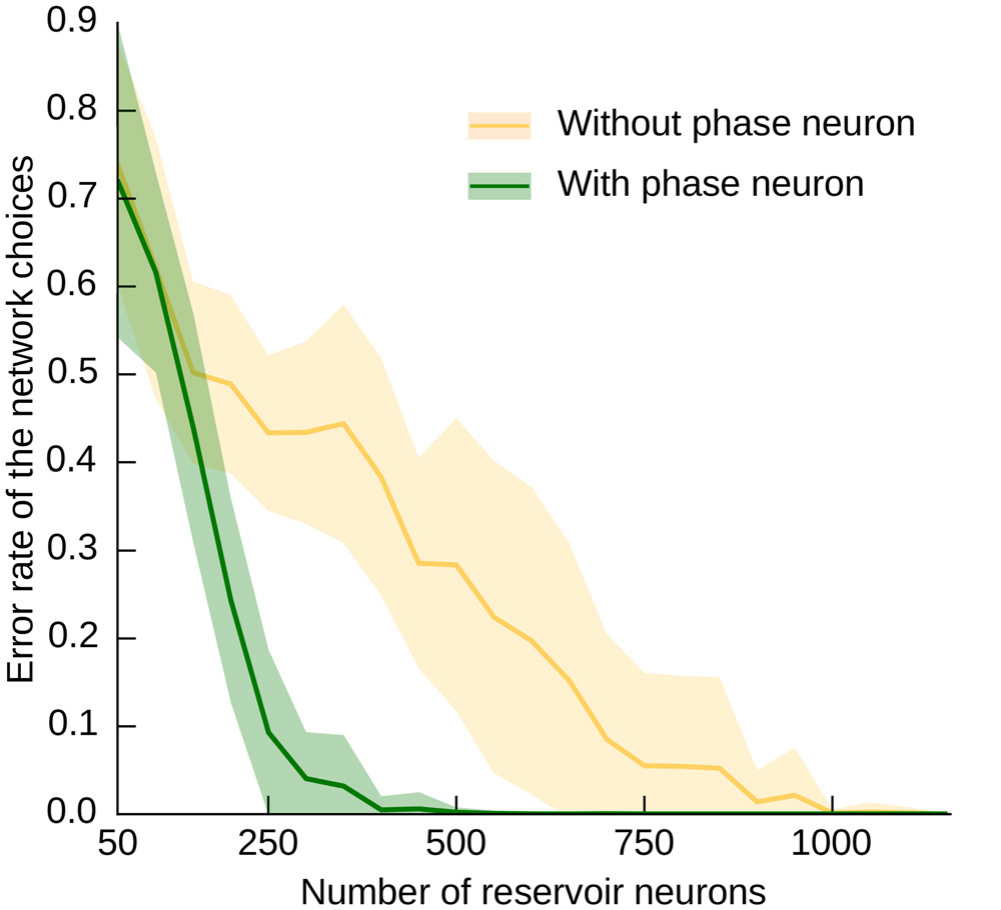
Error rates of the model as a function of the number of neurons in the reservoir (30 simulations, shaded area correspond to standard error). The model required less neurons to perform well when phase information was explicitly represented in the trained context neuron. Error rates were computed as the ratio between the number of suboptimal trials (choices that likely postpone the reward) over the total number of trials. The reservoir required ~400 neurons to perform the task optimally with phase information explicitly encoded in a readout neuron, vs. ~1000 neurons that were required without the context neuron.

Interestingly, feeding this explicit contextual information back into the reservoir greatly reduced the number of suboptimal choices made by the model. The explicit context version of the model performed very well with less than half the number of neurons in the reservoir originally required (Fig 9). The initial version of the model compensated for the lack of explicit contextual memory with a higher number of neurons that provided extended memory of the reward [40–42]. In the contextual memory version of the model, since phase information was explicitly encoded with the context neuron, neurons responding to the combination of phase and reward should be more numerous. Indeed, the percentage of neurons having a significantly higher activity for COR1 trials than for INC and COR trials was much higher with the context neuron feeding back phase information (13.2% ± 1.2%, p-value < 10^−15^), approaching the proportions seen in the primate.

While previous results illustrated the intrinsic capacity of reservoir network to encode complex combinations of previous and current inputs, here we demonstrate how information already present in the network can become explicitly encoded and amplified through learning. As a consequence, this strengthening of the internal representation allowed for combinations of internal and external inputs to be more widely represented in single unit activities, revealed by the increase in COR1 neurons, and for a significant performance increase, revealed by the reduced number of neurons required to solve the task.

We also tested the effects of noise on the coding of mixed selectivity in the presence of the phase neuron. Table 3 shows the percentage of neurons with different main effects and interactions, in networks with and without the Phase neuron. Presence of the phase neuron yields an increase in the mixed selectivity (interactions).

**Table 3:** Results of a three-way ANOVA (Epoch x Phase x Choice, FDR correction on all tests, all neurons, all simulations, p-value < 0.05) on reservoir with and without the Phase neuron (average ± std out of 1000 neurons over 30 simulations with 1 SD Gaussian noise).

	Model (Phase neuron)	Model (no Phase)
	Average % of significant neurons	Average % of significant neurons
Phase	97.60 ± 0.49	88.54 ± 1.92
Choice	97.12 ± 1.05	95.45 ± 1.82
Epoch	99.92 ± 0.07	99.98 ± 0.05
Epoch * Phase	89.26 ± 1.63	83.69 ± 2.54
Epoch * Choice	83.31 ± 4.16	76.42 ± 6.61
Phase * Choice	93.71 ± 2.32	74.20 ± 6.96
Epoch * Phase * Choice	52.71 ± 9.36	14.66 ± 7.10

### Context is represented in attractors

Training the context neuron revealed that dynamic mixed selectivity can be used to generate stable meaningful output at the population level. The constant activation of the contextual phase neuron during search phase and its complete inactivation during the repeat phase indicates that the phase information is available in the reservoir population activity. Reinjecting the activity of this context neuron back into the reservoir creates a system that can operate in two distinct submodes of dynamics. Pascanu and Jaeger [17] develop a framework in which working memory is implemented by such mechanisms that are characterized as input driven attractors. Indeed, the principle of the learning algorithm used in this experiment (FORCE learning) is to converge on weights that shape attractors of the neural dynamics in order to produce stable and robust dynamics [20].

**Fig 10:**
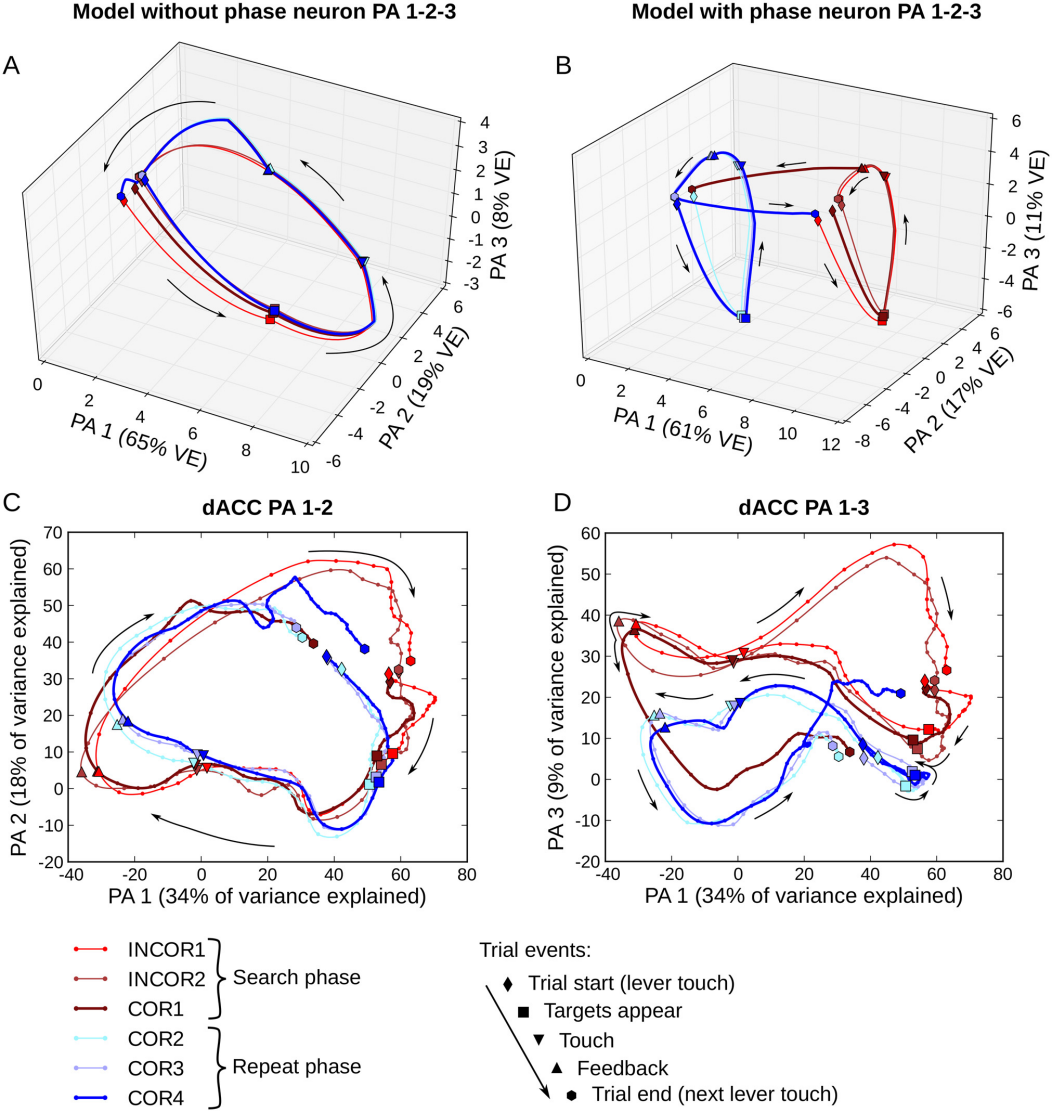
Reservoir and dACC population PCA-projected trajectories of successive trials in a problem show attractor-like dynamics. Trajectory colors represent trials of each phase, reddish colors for search and blueish colors for repetition. **A**. Model population trajectories of average activity without context neuron (dimensions 1, 2 and 3; 93% of variance explained). All trajectories show a similar cyclic shape showing the successive events of a trial. Search and repeat trajectories are only separated after feedback and seem to collapse into a single trajectory at the middle of the next trial. **B**. Same as **A** with context neuron version of the model (89% of variance explained). Search and repeat trajectories form two well separated cyclic trajectories resembling two distinct attractors. COR1 and COR4 trials were the transition trials between the two sets of trajectories. **C**. dACC neural population trajectories of average activity for successive trials of a problem on dimension 1 and 2 of the PCA (51% of variance explained with both dimensions). Points represent successive 100ms of averaged activity. Trajectories show a cyclic pattern representing the path of activity in a trial. Search and repeat trajectories seem to overlap mostly between target appearance and touch events. **D**. Same as **C** with dimension 1 and 3 instead (42% of variance explained). Search and repeat trajectories are well separated suggesting an attractor like encoding of phase at the population level. (PA: principal axis, VE: variance explained)

In order to reveal this specific dynamical property of the population activity we employed a PCA analysis to visualize the state trajectories of the reservoir neural population so as to assess whether search and repeat population activities were well separated. Full trial activities were averaged for each neuron of the reservoir population over each trial of a problem: INC1 and INC2 were the first and second unrewarded search trials (INC3 trials were ignored due to lack of trials), COR1 the first rewarded trial, COR2-4 the repeat trials with the presentation of the signal to change at the end of COR4 trial (see Methods for further details). Reservoir neuron population activity (without the context neuron) projected onto the first 3 dimensions shows the transient cyclic nature of all the trajectories, representing the common path of successive events in each trial (92% of explained variance over the first 3 dimensions, Fig 10A). Search and repeat trajectories were separated after feedback but then collapsed slowly and seemed to merge at the beginning of next trial. Since phase information was solely given by the reward in this original version of the model, trajectories were most separated after this event. While the trajectories tend to converge at the middle of the next trial, the phase information is sufficiently present to allow the model to successfully perform the task. We then performed the PCA on reservoir neurons in the explicit context version of the model. In this version of the model, phase information was explicitly encoded in the network dynamics as illustrated in the PCA results that show two well separated sets of cyclic trajectories representing the search and repeat phases (89% of explained variance over 3 first dimensions, Fig 10B). The signal to change at the end of a problem deviated the end of COR4 trajectory towards the start of search trials as the contextual phase neuron activation was triggered. Likewise, the first reward during COR1 trial acted as a signal for the transition from the search to the repeat trajectories as the context neuron shut off. Previous modeling and machine learning studies have shown how information representations could be maintained through attractors to develop a form working memory and participate in contextual processing of inputs [17, 43, 44]. These theories are supported by experimental results that also suggest rule representation and cognitive states through attractors [29, 45, 46].

Note that the dynamical regime of the reservoir population is more complicated than a single dimensional (point attractor) or limit cycle. As pointed out by Maass and colleagues [19], we are in the case of high dimensional attractors, whereby a few dimensions were caught in a attractor while the rest of the neural population dynamic was free of the attractor and still contributed to the real-time computing properties that are more generally associated with reservoirs. In our experiment, the attracting dimensions were the ones separating both loops, while the attractor free dimensions still involved in real-time spatio-temporal processing are seen in the loop pattern representing the successive events of the task. Furthermore, the strict definition of an attractor does not apply here, since the reservoir is constantly receiving new inputs and cannot be considered a closed system. This type of attractor, dependent on the inputs, has been termed input-induced attractors by Pascanu and Jaeger [17]. Our analyses support attracting dynamics as a plausible mechanism to implement contextual memory in neural networks to represent contextual information [17, 29].

We performed the same PCA analysis with dACC data to determine if dACC population activity displayed similar dynamics by comparing the qualitative features of the trajectories with those of the model. In a similar manner, each neuron’s activity was averaged over all the trials of the same type (a subset of 184/546 selected dACC neurons was used, see Methods). Projection of the dACC population activity onto the first two dimensions of PCA (53% of variance explained over 2 first dimensions, Fig 10C) revealed cyclic trajectories representing the common path for all trials, akin to the loop pattern observed in the model. However, these dimensions displayed slight trajectory differences between search and repeat trials, especially around feedback. This separation between search and repeat phase was much stronger with dimension 3 (43% of variance explained variance over dimension 1 and 3, Fig 10D). dACC neural population activity differed most after feedback and until the end of the trial which is explained by the presence of a reward only in the repeat trials. Yet, trajectories were still separated between the start of a trial and the feedback. This suggests that the population dynamics actively maintained separate states underlying behavioral context through attractors. As with the model trajectories, COR1 and COR4 trials displayed clear transitions between search and repeat trajectories.

These results support the claim that a reservoir with feedback connections is able to create a hybrid dynamical regime within a recurrent network, mixing attracting and transient dynamics, to extend the processing capabilities of a recurrent network beyond the exclusive attracting or transient regimes largely explored in the literature. dACC analyses strongly suggest the presence of an attractor to represent phase, while showing a globally dynamic activity thereby constituting a proof of concept of reservoir computing and its hybrid dynamical regime in a prefrontal area.

**Fig 11:**
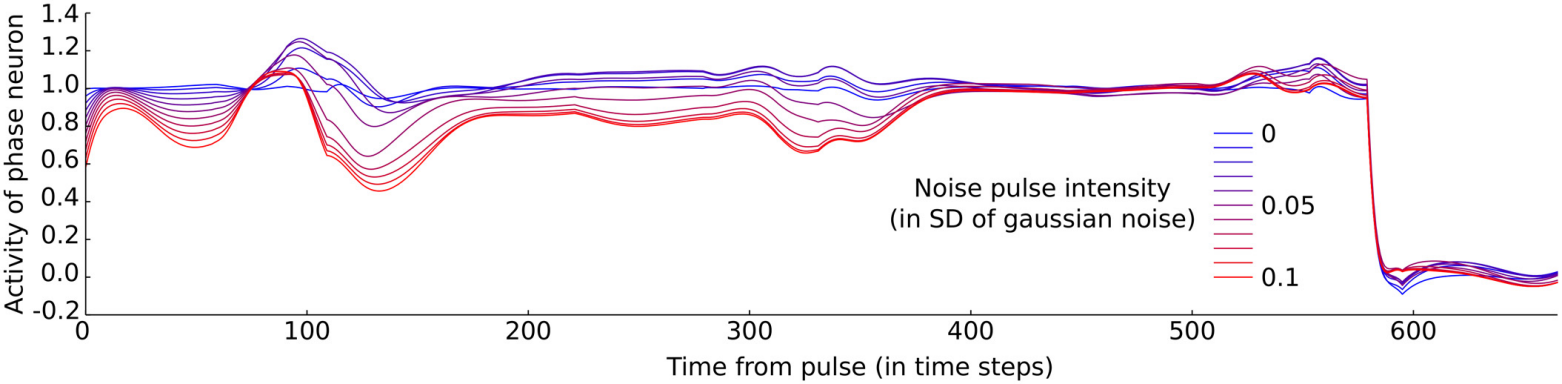
Activity of phase neuron in response to perturbation. Noise pulses were sent to the network at the beginning of the same INC1 trial. Gaussian noise was injected directly in the neurons (in the membrane potential) and not in the input (as was the case for the mixed selectivity noise simulations). Simulation after the noise pulse lasted 3 trials. Noise pulses ranged from 0 SD (no pulse) to 0.1 SD with increments of 0.01 SD using the same seed so that every pulse are scaled versions of a unique pulse. Each line represents the activity of the phase neuron for one level of noise, color coded from blue (0 perturbation) to red (maximum perturbation).

If we hope to better understand computation in recurrent cortical networks in terms of computation in reservoirs, then we should first better understand what is happening in these reservoirs. The high dimensional projection of inputs and previous states in the reservoir provides a vast repertoire of states that can be selected for the task at hand by training the readouts. In order to understand how the neural dynamics implement system behavior Sussillo et al. [47] developed methods that revealed how trained recurrent neural networks solved tasks by creating attractors (in this case fixed points) that represent memory states, and saddle points that transition between the attractors as required for the task. In our case, we demonstrate that in an untrained reservoir the information related to the task phase (search vs. repeat) is reliably coded in the network population. When we then create a feedback neuron that is trained to explicitly represent this information, we in effect create an input dependent attractor that allows the network to solve the task with half the number of neurons. Thus, similar to the input driven working memory units in [17], our phase neuron corresponds to a task dependent input-induced attractor that is created by the network in order to solve the problem. Thus, already within untrained networks, the high dimensional projection creates representations required for solving a class of problems. Adaptation can isolate such representations and make them explicit and thus optimizing the network for the task. The attractor properties of these representations can be illustrated by showing how its activity is stable to perturbations. Fig 11 displays the stability of the phase neuron in response to noise pulses of different intensities.

## Discussion

Rigotti et al. [2] recently postulated that randomly connected networks could account for a form of universal combinations of inputs to the system. One of the principal characteristics of these networks is the mixed selectivity to task-related factors that is observed in single neurons. We first observed such responses as a mix of target location and sequence rank responses in neurons of a reservoir model trained in a motor sequencing task [8], as had been observed in primate PFC [4]. This same mixed selectivity in single cortical units is also crucial for the successful performance of these tasks by trained primates [7]. However, in many tasks involving executive function, the context provided by previous stimuli will be required for meeting current behavioral demands. Past inputs that influence future behavior create a context that forms a temporal bridge between past events and the current situation [48]. So, context should be internally represented and contribute to the mixed selectivity combinations and universal representation of contingencies that will include past events. Interestingly, random recurrent networks like reservoirs combine current inputs with current states resulting from previous inputs. Consequently, in addition to its inherent ability to generate mixed selectivity, the reservoir computing (RC) paradigm provide an hypothesis on how temporal information can be combined with mixed selectivity to generate a dynamic form of mixed selectivity. The present study demonstrates the presence of dynamic mixed selectivity in a complex cognitive task, both in a reservoir and in a prefrontal cortical area.

Because of their complexity, dynamic mixed selectivity activities do not lend themselves to straightforward interpretations. However, in the RC paradigm, they are the signature of a high dimensional projection resulting from the combination of current inputs (spatial information) and previous inputs and their order (temporal information), which is characteristic of what has been termed spatiotemporal processing in neural networks [37]. Recurrent networks are particularly suited to carry out spatiotemporal processing. Recognizing that the random connectivity in recurrent networks itself provides rich spatiotemporal representations, the RC paradigm eliminates the training of the recurrent connections and demonstrates that computational power is inherently present in this type of network. Given that our RC model reproduced qualitative features of the dynamic mixed selectivity observed in dACC, we support the hypothesis that local recurrent connectivity in the cortex may be at the origin of this representational feature. A corollary of this hypothesis is that mixed selectivity should be present before learning a new task [2, 9]. Further experiments recording activity in prefrontal areas could demonstrate the presence of mixed selectivity prior learning, as shown in other parts of the cortex [13, 15].

As in any neural network model, simulating a local cortical network in isolation of external perturbations elicited extremely stable activities in our reservoir compared to the recorded dACC data. Adding noise to the model during training and testing reduced the otherwise high mixed selectivity values. As predicted, performances decreased rapidly with reduced mixed selectivity, and the more complex selectivity (i.e. the 2 and 3 way ANOVA interactions) decreased more than simple selectivity (i.e. ANOVA main effects), leaving the model with a proportion of more complex selectivity gradually lower than simpler ones. Interestingly, proportions of selectivity in the dACC data was similarly ordered in decreasing complexity.

Because the reservoir networks have a fixed connectivity, their modeling power lies in the inherent capacity of random recurrent networks to produce rich combinations of inputs. Yet learning in monkeys most certainly influences local network connectivity, therefore, learning might have strengthened already present mixed selectivity to elicit robust representation in the face of noise. To illustrate how learning may have influenced representations, we explored the representation of the first reward in a problem (COR1 reward), which indicated the crucial switch from exploration to exploitation behavior. dACC neurons displayed a strong and specific activity at the time of the first reward. Our model with fixed connections seem to present only slight differences in the activity of only a few COR1 neurons which may be attributed to chance. However, training the network to represent this information in a manner similar to the dACC COR1 activities demonstrates that this information, while not robustly present in the activity of single units, can robustly be extracted from the dynamic mixed selectivity of the population, and represented explicitly. Learning may be the mechanism by which inherently but weakly represented information may be extracted to be represented explicitly.

Explicit representation has several non-negligible benefits. Locally, it increases the robustness of the represented information, adding an internally generated variable that might be further combined with other internal variables and inputs, thereby expanding the representational power of the local network. In addition, long range communication may benefit from explicitly represented information which relies on more condensed representations and could save bandwidth. COR1 activities are sharp and transient, acting putatively as a detector of a particular contingency. A putative local role of the COR1 activity might be to robustly trigger the transition from the search to repeat attractors while signaling impending change in behavioral demand to more distal areas. This type of universal coding that is then shaped by learning could be a key feature of primate adaptation. A developmental form of this universal coding and subsequent adaptation has been observed for the vowel space in the auditory system that is initially universal and then focused to the native language in early development [49].

Reservoir models also provide insights on the dynamics of the population. Since the recurrent network connectivity in RC is fixed and generated according to only a few parameters suited for the task at hand, it elicits generic dynamics that are of interest to understand the inherent dynamics that might be generated in a local cortical network. The autocorrelation results illustrate this point: dynamics in the model tend to closely follow the sequence of inputs fed to the network and appeared to reproduce the major features of the dynamics in the dACC, leading to two conclusions. First, the population activity in the model as in the dACC was transient (dynamic) over the course of full trials. Secondly, as mentioned above, the shape of the autocorrelation pattern tended to follow the course of events in the task, in both model and dACC. The dACC autocorrelation pattern could also be explained by an increase in activity changes resulting from event processing mechanism appearing thanks to learning the task, which would also coincide with task events, but would not be a result of local cortical network connectivity. However, our results demonstrate that these dynamic patterns are inherently present in recurrent networks, which do not support this alternative hypothesis as a unique explanation and, on the contrary, suggest dynamics inherent to the recurrent connectivity, that could possibly be modified with learning.

Since the population dynamic as a whole was transient it is unclear how stable information could be robustly encoded throughout complete trials. However, a key principle of RC is computing without stable states, where high dimensional and transient activity is elicited by the recurrent network while a linear readout separate states of interest, in a manner similar to the state of the art SVM [50–52]. Indeed, the reservoir could continuously represent the two states of the task phase (search vs. repeat) via a linear readout even though the population activity is globally transient and no single neuron represented this variable. Similarly, phase was steadily decoded from dACC population activity and revealed a sharp transition between the two task phases. Phase information was crucial in this task to adjust behavior, and may explain the numerous neurons sensitive to phase in dACC. Indeed, adding a feedback connection to a readout neuron explicitly representing phase drastically reduced the number of reservoir neurons necessary to perform the task, emphasizing the importance of coding this variable in this task. Indeed, in the absence of explicit representation, the network relied on its fading memory (which depends on the number of neurons) of the reward input to access phase information. Explicit representation of task phase in a feedback loop created an attractor that maintained the state of the phase variable. Yet, no single dACC neuron was found to continuously discriminate behavioral phase with an ON or OFF state as was the case with the model, and improvements of our implementation of the phase representation may yield interesting insights into the nature and origin of long-lasting distributed transient contextual representations through attractors.

Pascanu and Jaeger [17] addressed this issue, training working-memory (WM) neurons to enter on or off states depending on particular inputs. Activation of these WM neurons shifted the network into a new subspace of dynamics, effectively generating input driven attractor states. This is analogous to our situation with the phase neuron. The learned on- or off-state of the phase neuron puts the reservoir in two distinct respective states, corresponding to the behavior appropriate for the search vs. repeat phases, respectively. We can thus consider that our results provide a concrete neurophysiologically grounded example of the input-driven attractor defined by Pascanu and Jaeger.

PCA visualization of the population activities illustrated how explicit representation of the phase variable separated trajectories of activities in distinct parts of the state space that were otherwise very similar. The same analysis on dACC data supports the hypothesis that the phase variable was maintained through attractors as well. Though the separation of the distinct phases was not as distinct, trajectories were separated at all times, as confirmed by the linear decoding of phase. Both model and dACC might be considered as dynamical systems switching between two attractors representing internal states. Attractors have been proposed as a candidate mechanism underlying robust information maintenance in working memory [53, 54], and also for decision making [55]. While autocorrelation emphasized a transient population dynamic, reservoir feedback mechanisms and PCA support the hypothesis of attracting states. Our results demonstrate how these seemingly incompatible dynamical regimes can actually coexist in neural dynamics. Indeed, Jaeger and Maass have both demonstrated that reservoir networks can create attracting dynamics while transient activities more familiar to this type of networks are still present [17, 19]. The type of attractors described here has been termed input driven attractors by Jaeger because they are triggered by a combination of internal states and inputs. In our case, it corresponds to the current state of the phase and the presence/absence of the reward input. Maass has described these as high dimensional attractors since they span only a few dimensions of the state space, thereby leaving other dimensions free to carry on spatiotemporal processing [19]. This hybrid dynamical regime provides high computational power, exceeding the capacities of systems relying purely on attractor or transient dynamics. While transient dynamics are inherent to a fixed recurrent network, attracting dynamics adapted to the task at hand may only appear with learning for explicit maintenance of task relevant information.

The comparison between reservoir and dACC activity can inform us about the nature of dACC function. The reservoir integrates sensory inputs including reward feedback, and determines appropriate responses based on the behavioral context. The context is determined based on the reward feedback. This is consistent with our understanding of dACC function, as an area that integrates outcomes to generate internal states that determine appropriate behavior. This is in contrast to related prefrontal areas such as DLPFC that receive input from dACC and are closer to the choice selection and behavioral response generation [23].

The use of such network studies to understand cortical function is becoming increasingly pertinent. In an effort to understand prefrontal cortical neurophysiology of working memory, Barak et al. [56] consider three models that define a functional transition between a form of pre-specified network, a recurrent network whose internal structure adapts to the structure of the stimuli, and a recurrent reservoir network. They observe that in accounting for the PFC data from a working memory task, it is the recurrent model whose internal structure adapts to the task that best accounts for the data. Rigotti et al. [57] similarly demonstrate how learning within a recurrent network allows for the creation of new attractors that can become the building blocks for representing task relevant context. Recently, Saez et al. [58] found evidence for the coding of context in a task where behaving primates frequently had to shift between two distinct behavioral contexts. Because of the arbitrary nature of the task, these context encoding neurons could only have emerged through the animals’ adaptation to the task. This is consistent with the proposal that intrinsic capabilities to code task relevant context, implemented in recurrent connections, can be captured and enhanced as needed through learning, as observed by Sussillo and Abbott [20], and Pascanu and Jaeger [17], and illustrated here with our phase context neuron.

The results reported in this work are proposed as a proof of concept of the reservoir computing (RC) paradigm as a model of representational and dynamical properties of local generic prefrontal cortical networks in a cognitive task. Strong local recurrence is a striking property of the cortex, and constitutes the main architectural feature modeled by a reservoir. Here we demonstrated that an RC architecture can perform a complex cognitive task, and that certain characteristic electrophysiological features and dynamical patterns of activity of dACC neurons were replicated. We argue that the similarities in activity patterns between the dACC and RC model are the result of their common recurrent architecture, emphasizing its role in information representation schemes in the PFC. While mere random recurrent connectivity has been shown to provide universal spatio-temporal processing capabilities to RC networks [9], their distributed mixed-selectivity representations may need to be strengthened and their inherent fading memory may restrict their processing capabilities in time. In this context, the role of task-learning might be to reinforce already present weak yet relevant signals, and to robustly extend the time-limited influence of previous state through time via input driven attractors [17, 20].
